# Venous thromboembolism, chronic liver disease and anticoagulant choice: effectiveness and safety of direct oral anticoagulants versus warfarin

**DOI:** 10.1016/j.rpth.2023.102293

**Published:** 2023-12-09

**Authors:** Oluwadolapo D. Lawal, Herbert D. Aronow, Anne L. Hume, Fisayomi Shobayo, Kelly L. Matson, Marilyn Barbour, Yichi Zhang, Xuerong Wen

**Affiliations:** 1 Department of Pharmacy Practice, College of Pharmacy, University of Rhode Island, Kingston, Rhode Island, USA; 2 Lifespan Cardiovascular Institute, Providence, Rhode Island, USA; 3 Division of Cardiology, Warren Alpert Medical School of Brown University, Providence, Rhode Island, USA; 4 Department of Cardiology, University of Texas Health Science Center, Houston, Texas, USA; 5 Department of Computer Sciences and Statistics, University of Rhode Island, Kingston, Rhode Island, USA

**Keywords:** anticoagulants, factor Xa inhibitors, liver diseases, venous thromboembolism, warfarin

## Abstract

**Background:**

Little to no data exist to guide treatment decision in patients with venous thromboembolism (VTE) and chronic liver disease.

**Objectives:**

To assess the effectiveness and safety of direct oral anticoagulants (DOACs)—individually and as a class—vs warfarin and between 2 DOACs in patients with acute VTE and chronic liver disease.

**Methods:**

We conducted a retrospective, US claims–based, propensity score–matched cohort study in adults with acute VTE and chronic liver disease who had newly initiated oral anticoagulants between 2011 and 2017. The primary outcome was a composite of hospitalization for recurrent VTE and hospitalization for major bleeding.

**Results:**

The cohorts included 2361 DOAC-warfarin, 895 apixaban-warfarin, 2161 rivaroxaban-warfarin, and 895 apixaban-rivaroxaban matched pairs. Lower risk of the primary outcome was seen with DOACs (hazard ratio [HR], 0.72; 95% CI, 0.61-0.85), apixaban (HR, 0.48; 95% CI, 0.35-0.66) or rivaroxaban (HR, 0.73; 95% CI, 0.61-0.88) vs warfarin but not apixaban-rivaroxaban (HR, 0.68; 95% CI, 0.43-1.08). The HRs of hospitalization for major bleeding were 0.69 (95% CI, 0.57-0.84) for DOAC-warfarin, 0.43 (95% CI, 0.30-0.63) for apixaban-warfarin, 0.72 (95% CI, 0.58-0.89) for rivaroxaban-warfarin, and 0.60 (95% CI, 0.35-1.06) for apixaban-rivaroxaban. Recurrent VTE risk was lower with apixaban (HR, 0.47; 95% CI, 0.26-0.86), but not DOACs (HR, 0.81; 95% CI, 0.59-1.12) or rivaroxaban vs warfarin (HR, 0.81; 95% CI, 0.57-1.14) or apixaban-rivaroxaban (HR, 0.92; 95% CI, 0.42-2.02).

**Conclusion:**

While the magnitude of clinical benefit varied across individual DOACs, in adults with acute VTE and chronic liver disease, oral factor Xa inhibitors (as a class or individually) were associated with lower risk of recurrent VTE and major bleeding.

## Essentials

• Little data exist on anticoagulation in patients with venous thromboembolism and liver disease.
• We sought to answer this important clinical question using data from routine clinical care.
• The primary outcome was hospitalization for recurrent venous thromboembolism or major bleeding.
• The risk of the primary outcome was lower with direct oral anticoagulants than with warfarin.

## 1 Introduction

Venous thromboembolism (VTE) is the third most common vascular disease in the United States [[1], [2], [3]]. VTE is estimated to affect up to 1 million Americans each year [[1], [2], [3]] and is implicated in the death of nearly 100,000 Americans annually [4].

Direct oral anticoagulants (DOACs) comprising oral factor (F)Xa inhibitors (ie, apixaban, rivaroxaban, and edoxaban) and direct thrombin inhibitors (ie, dabigatran) are the first-line treatment option in the management of VTE [[5], [6], [7]]. Anticoagulation reduces the risk of recurrent VTE and death, which are highest within the first 3 to 6 months of an index VTE event. While the utility of DOACs compared to warfarin has been investigated in patients with complex comorbid conditions such as chronic kidney disease [8,9] and active cancer [10], clinical trial and real-world data in individuals with VTE and chronic liver disease are scant [8,11]. Individuals with elevated liver enzyme levels and chronic liver disease were excluded from the approved clinical trials of DOACs for VTE treatment [[12], [13], [14], [15]], and so far, there are no clinical trials comparing DOACs and warfarin in patients with VTE and chronic liver disease. There is also no large, population-based study of DOACs compared with warfarin, between 2 DOACs in patients with VTE and chronic liver disease, or among patients with VTE and severe chronic liver disease. Available real-world data on the risk-benefit profile of DOACs in patients with VTE and chronic liver disease are mainly from case series with fewer than 100 DOAC-exposed subjects [8,11,16,17]. These smaller studies are, however, underpowered to detect meaningful clinical differences between DOACs and warfarin and have limited generalizability to routine clinical care.

Of note, DOACs rely, to varying degrees, on the liver for hepatic clearance (75% for apixaban, 65% for rivaroxaban, 50% for edoxaban, and 20% for dabigatran) [8]. Therefore, elevated levels of DOACs in the blood arising from impaired hepatic dysfunction may pose further harmful effects in patients requiring oral anticoagulants. The risk of thrombotic events and bleeding complications is also significantly increased in patients with chronic liver disease [8,11]. Considering the increasing adoption of DOACs to manage VTE coupled with the increasing incidence of chronic liver disease in the United States and shared risk factors between VTE and chronic liver disease [8,11], there is a need for high-quality real-world data to guide treatment decisions between DOACs and warfarin in this high-risk population. Accordingly, we evaluated the magnitude of clinical benefit of DOACs (individually and as a class) vs warfarin and between DOACs in patients with VTE and chronic liver disease.

## 2 Methods

### 2.1 Data source

This retrospective real-world cohort study was performed using administrative claims submitted to the Optum Clinformatics Data Mart database from January 1, 2010, through December 31, 2017. The Optum database contains deidentified, nationally representative data of ≥20 million enrollees of commercial and Medicare Advantage health insurance plans across the United States. Information on beneficiaries includes demographic data, hospitalization and outpatient visits, laboratory data (in a small subset of enrollees), and outpatient prescription services. This study was approved by the University of Rhode Island Institutional Review Board; the requirement for informed consent was waived due to the anonymous nature of the Optum data.

### 2.2 Study population

The study population comprised individuals aged ≥18 years with a diagnosis of chronic liver disease who initiated DOACs (apixaban, rivaroxaban, edoxaban, or dabigatran) or warfarin between January 1, 2011, and December 31, 2017, after the first diagnosis of VTE [18]. The date of first fill of oral anticoagulant was regarded as the index date, and the 1-year period prior to the index date was regarded as the baseline period. While the database included data from January 2010 through December 2017, given the new-user design and 1-year baseline period, the first possible date of patient inclusion was January 1, 2011. VTE was defined using validated algorithms, defined as ≥1 inpatient or outpatient claim for acute deep vein thrombosis (DVT) or pulmonary embolism (positive predictive value [PPV], >90%; Supplementary Table 1) [[18], [19], [20]]. Chronic liver disease was identified using diagnosis codes for conditions associated with prolonged or complete deterioration of liver function in any position in inpatient or outpatient claims (Supplementary Table 1)[21,22].

Eligible patients were required to have (a) filled a single oral anticoagulant on the index date, (b) ≥12 months of continuous medical and pharmacy insurance eligibility with no more than a 30-day gap before the index date, (c) initiated oral anticoagulants within 30 days of hospital discharge (if inpatient) or service date (if outpatient visit) for first VTE diagnosis [18], and (d) health encounters for chronic liver disease within 12 months of the index dispensing (Supplementary Figure 1). We required filling of oral anticoagulants within 30 days of health encounter for index VTE to increase the likelihood that the source population comprised patients who initiated oral anticoagulation for management of VTE. A 30-day period between index VTE diagnosis and the index date was required to ensure patients had a reasonable amount of time to fill and initiate outpatient oral anticoagulants after index VTE diagnosis.

We excluded patients with medical encounters for alternative indications for oral anticoagulation (eg, atrial fibrillation, mitral stenosis, valve repair, and replacement), pregnancy, cesarean section, prior bleeding (to capture incident bleeding events after index VTE), dialysis or kidney replacement, and inferior vena cava filter during the baseline period. Based on the index oral anticoagulant fill, eligible subjects were categorized as either DOAC or warfarin users.

### 2.3 Outcomes

The clinical outcomes, identified using validated algorithms, were analyzed both separately and as a composite. The primary outcome measure was the net clinical benefit, a composite of hospitalization for recurrent VTE and hospitalization for major bleeding. Recurrent VTE, the primary effectiveness outcome, was defined as primary diagnosis in inpatient claims for acute DVT or pulmonary embolism >7 days after index VTE event (PPV, ≥89%; Supplementary Table 2) [[18], [19], [20],^23^]. Relatively poor PPVs have been reported with algorithms for VTE based on events that occurred in outpatient settings or defined by the presence of primary or secondary diagnoses. Therefore, eligible recurrent VTE events were restricted to those that occurred in inpatient settings and reported as the primary diagnosis for hospitalization [24]. Major bleeding, the primary safety outcome, was defined by the presence of transfusion codes or primary diagnosis for bleeding in inpatient claims (PPV, ≥89%; Supplementary Table 2) [23,25,26]. All-cause mortality and clinically relevant nonmajor bleeding (CRNMB) were the secondary effectiveness and safety outcomes, respectively. All-cause mortality, available in the database, was obtained by linkage of the database with the Social Security Administration Death Master File. The specificity of mortality status in the Death Master File has been reported to be ≥97% [27,28]. Due to changes in reporting requirements of mortality data by individual US states to the Social Security Office, death data in the Optum Clinformatics database may be incomplete from 2013 onwards [[27], [28], [29]]. CRNMB was defined by secondary diagnosis for bleeding in inpatient claims or primary or secondary diagnosis for bleeding in outpatient claims [23,25]. We distinguished between hospitalization for major bleeding and CRNMB to align with pivotal DOACs for VTE clinical trials where these safety outcomes were captured separately [[12], [13], [14], [15]], to more closely align with International Society on Thrombosis and Haemostasis definitions for major bleeding and CRNMB [30,31], and to differentiate between the severity of bleeding events. The safety outcomes were collectively referred to as clinically relevant bleeding.

### 2.4 Follow-up

Subjects were followed in an as-treated manner the day after the index date for up to 183 days until first diagnosis of an outcome, switch to comparator drugs, treatment discontinuation (defined as a gap of >30 days after the end of days’ supply of the previous and subsequent fill), insurance disenrollment, or study end date, whichever came first. We chose a primary follow-up time of up to 183 days for outcome assessment to align with the pivotal trials for VTE [[12], [13], [14], [15]] and because the recommended treatment duration for primary treatment of acute VTE is 3 to 6 months [7].

### 2.5 Covariates

Several known and suspected risk factors for the outcomes of interest were adjusted for our analyses. These baseline covariates were broadly categorized into (a) demographic characteristics, (b) type of index VTE event, (c) comorbid conditions and lifestyle factors, (d) concomitant drug use (eg, P-glycoprotein inhibitors) [32,33], (e) risk score for bleeding (ie, the Hypertension, Abnormal renal/liver function, Stroke, Bleeding history or predisposition, Labile international normalized ratio, Elderly (>65 years), Drugs/alcohol concomitantly score [HAS-BLED] score) [34] and (f) a claims-based frailty index reported by Kim et al. [35] to further adjust for burden of illness. A complete list of the covariates and time of assessment is presented in Supplementary Table 3. Balance on the frequency and results from relevant serologic tests were also assessed in the subgroup of subjects where this information was available (*n* = 1853); these variables were, however, not adjusted for in the analysis models.

### 2.6 Statistical analysis

**Figure 1:**
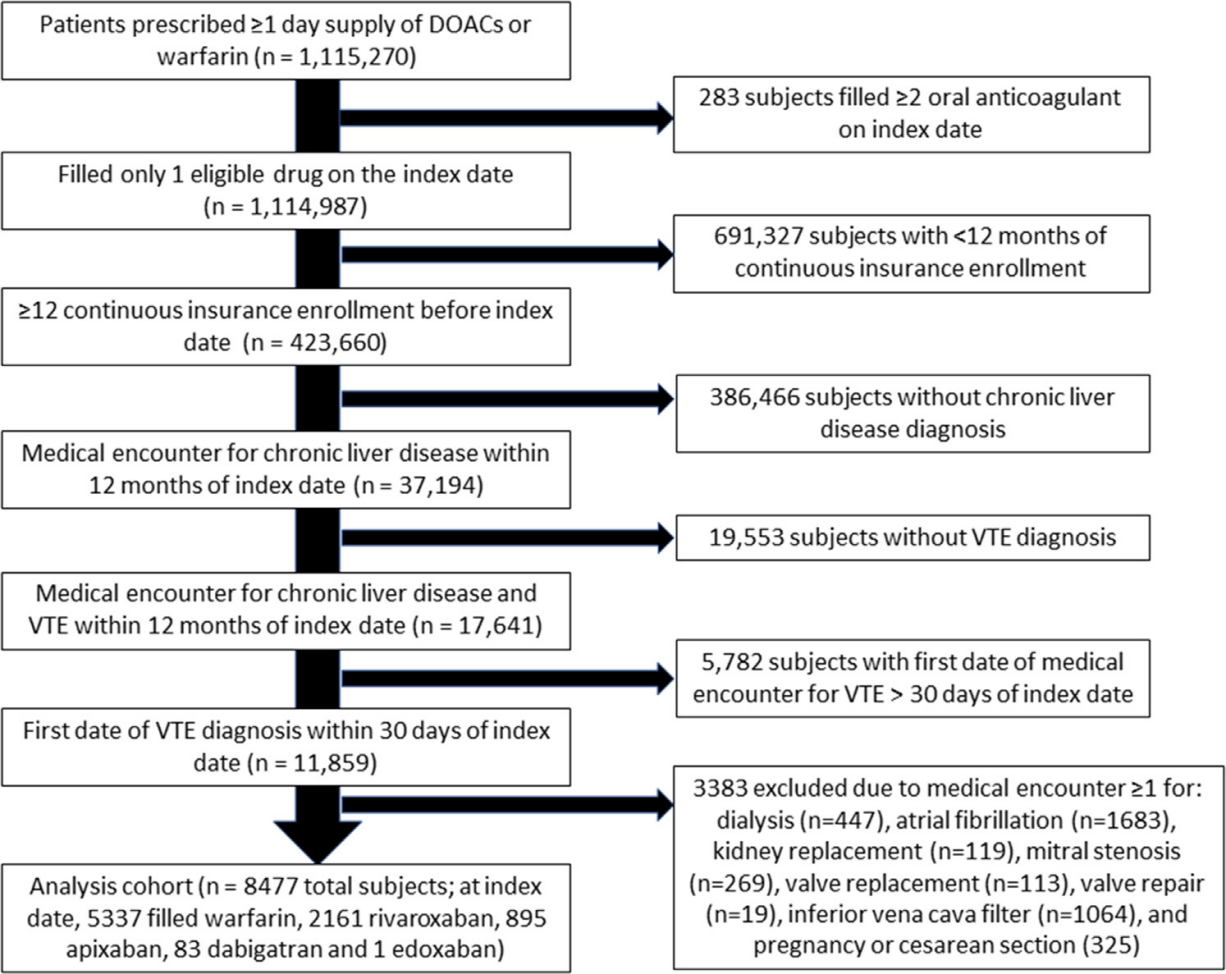
Study selection. DOAC, direct oral anticoagulant; VTE, venous thromboembolism.

Propensity score (PS) methods were used to balance the treatment groups on measured baseline covariates. The PS for being in a treatment group was estimated using a logistic regression model and corresponds to the predicted probability of receiving treatment given the baseline covariates. DOAC and warfarin users were then matched in a 1:1 ratio (by nearest neighbor without replacement) using a caliper width equal to 0.2 of the SD of the logit of the PS [36] Balance between the 2 treatment groups after PS matching (PSM) was assessed using standardized differences, with a threshold of ≤0.1 to imply the absence of substantial imbalance [36]. Further, we assessed differences in risk for clinical outcomes by exposure to a specific DOAC vs warfarin and for head-to-head DOAC comparison. Based on the index fill, matching was repeated to yield cohorts comparing (a) apixaban vs warfarin, (b) rivaroxaban vs warfarin, and (c) apixaban vs rivaroxaban. The exposure to specific DOACs vs warfarin and DOAC-DOAC comparisons were limited to oral direct FXa inhibitors users (ie, apixaban and rivaroxaban) as they accounted for 97% (*n* = 3056) of the 3140 subjects that initiated DOACs (Figure 1).

The cumulative incidence rate (IR) of the study outcomes was illustrated using Kaplan–Meier curves, compared using a stratified log-rank test, and presented as the total number of events per 100 person-years. After PSM, hazard ratios (HRs) and their corresponding 95% CI were estimated using Cox proportional hazard regression models, with a robust variance estimator to account for correlation between matched subjects. The proportional hazard assumption was assessed by visual inspection of the log-log survival curves and testing the Schoenfeld residuals. We did not observe evidence of violation of the proportional hazards assumption for any outcomes.

### 2.7 Secondary and subgroup analyses

We assessed the net clinical benefit of DOACs vs warfarin in individuals with VTE and severe chronic liver disease, either cirrhosis or its advanced stage decompensated cirrhosis. The absence of the clinical components of the Child–Pugh scores and limited availability of laboratory tests hindered the computation of Child–Pugh scores (Supplementary Methods). Alternatively, cirrhosis and decompensated cirrhosis were identified using validated diagnosis-based algorithms (PPV, >78%-91%; Supplementary Table 1) [[37], [38], [39]]. Further, we assessed for the presence of effect modification of observed relationships between DOACs and warfarin by age categories, sex, and the presence of cancer, nonalcoholic fatty liver disease (NAFLD; because prior data suggest substantial drug metabolism alterations, particularly of cytochrome P450 3A4 in NAFLD) [8,40,41], chronic kidney disease, provoked or unprovoked VTE, and diabetes. Provoked VTE was defined as hospitalization for ≥3 days, receipt of estrogen therapy, or medical encounters indicative of trauma, fracture, surgery, or active cancer (presence of procedural codes for radiation therapy or chemotherapy) within 3 months of index VTE event [42].

### 2.8 Sensitivity analyses

First, to simulate the other treatment durations assessed in the clinical trials of DOACs for VTE treatment [[12], [13], [14], [15]], we repeated our analyses using an alternative follow-up period of up to 3 months or 12 months. Second, we repeated our analyses using an intent-to-treat approach to evaluate the possible effects of informative censoring, wherein subjects were followed from the index date till occurrence of a censoring event, without considering treatment discontinuation or switching. Third, to increase the likelihood that subjects remained anticoagulated during follow-up, we reduced the maximum allowable gap between fills to (a) a 7-day gap, (b) a 14-day gap, and (c) a gap based on the half-life for each oral anticoagulant [8,11] (Supplementary Methods). Fourth, to accommodate patients who entered the cohort toward the end of the study period while facilitating potential accrual of follow-up time, the primary analyses were repeated using a cohort restricted to patients who initiated treatment between January 1, 2011, and June 30, 2017. Fifth, using a Fine and Gray subdistribution hazard model, we adjusted for the competing risk of death in analysis of the primary outcome and the primary effectiveness and safety outcomes.

Finally, 3 sets of additional sensitivity analyses were conducted to assess potential unmeasured and substantial residual confounding. First, we performed high-dimensional PSM (hd-PSM) using prespecified covariates and 200 additional empirically identified covariates that may serve as proxies for other unmeasured confounders [43]. Second, we calculated an E-value to assess the strength of an unmeasured confounder needed to fully explain away the exposure-outcome association, conditional on measured confounders [44]. Third, to exclude the presence of substantial residual confounding, we performed a negative control analysis using pneumonia as the outcome measure [45], an outcome with no known relationship that differs between DOACs and warfarin.

All analyses were conducted using SAS 9.4 (SAS Institute). No adjustments were made for multiple comparisons.

## 3 Results

### 3.1 Baseline characteristics

Before PSM, 8477 subjects, with 5337 (63%) initiating warfarin and 3140 (37%) initiating DOACs, were eligible for inclusion (Figure 1). Rivaroxaban (2161; 68.8%) and apixaban (895; 28.5%) were more frequently filled DOACs; 83 subjects filled dabigatran and 1 edoxaban. Of the 8477 subjects, 5927 discontinued treatment, while 505 subjects switched treatment from rivaroxaban (245) or apixaban (245) to warfarin, the most common (Supplementary Table 4). At baseline, majority (4383; 51.7%) of the 8477 subjects were women, with a median age of 65 years (IQR, 55-73 years), and more commonly had diagnoses indicative of provoked VTE (6471; 76.3%) (Table 1, Supplementary Table 5). The index VTE type was mostly DVT (5454; 64.3%), with the remaining cases being pulmonary embolism (Table 1, Supplementary Table 5). NAFLD (2832; 33.4%), cirrhosis (2449; 28.9%), and viral hepatitis (685; 8.1%) were the most common chronic liver disease etiologies (Supplementary Table 6).

**Table 1:** Select baseline characteristics of patients with acute venous thromboembolism and chronic liver disease initiating direct oral anticoagulants vs warfarin before and after propensity score matching.

Characteristics^a^	Before PSM, subjects *n* (%)			After PSM, subjects *n* (%)		
	DOAC users (*n* = 3140, 37.0%)	Warfarin users (*n* = 5337, 63%)	Std diff.	DOAC users (*n* = 2361, 50%)	Warfarin users (*n* = 2361, 50%)	Std diff.
Demographic characteristics						
Age (y), mean (SD)	63 (13.5)	63.6 (13.7)	−0.05	63.8 (13.3)	64.1 (13.7)	−0.02
Age category, ≥65 y	1529 (48.7)	2724 (51)	−0.05	1206 (51.1)	1238 (52.4)	−0.03
Female	1607 (51.2)	2777 (52)	−0.02	1217 (51.5)	1224 (51.8)	−0.01
Type of index VTE episode						
DVT	1977 (63.0)	3477 (65.1)	−0.05	1512 (64)	1506 (63.8)	0.01
PE	1163 (37.0)	1860 (34.9)	0.03	1086 (46)	1091 (46.2)	0.00
Comorbid conditions and lifestyle factors						
Hypertension	2231 (71.1)	3874 (72.6)	−0.03	1699 (72)	1702 (72.1)	0.00
Diabetes	1048 (33.4)	1914 (35.9)	−0.05	778 (33)	808 (34.2)	−0.03
Hyperlipidemia	1795 (57.2)	3072 (57.6)	−0.01	1336 (56.6)	1344 (56.9)	−0.01
Chronic renal disease	559 (17.8)	1005 (18.8)	−0.03	434 (18.4)	455 (19.3)	−0.02
Provoked VTE	2228 (71)	4243 (79.5)	−0.20	1721 (72.9)	1782 (75.5)	−0.06
Cancer	1596 (50.8)	2715 (50.9)	0.00	1189 (50.4)	1166 (49.4)	0.02
Medication history						
Statins	1209 (38.5)	2066 (38.7)	0.00	907 (38.4)	913 (38.7)	−0.01
Metformin	481 (15.3)	844 (15.8)	−0.01	348 (14.7)	358 (15.2)	−0.01
NSAIDs	897 (28.6)	1375 (25.8)	0.06	634 (26.9)	623 (26.4)	0.01
COX-2 inhibitors	93 (3)	165 (3.1)	−0.01	71 (3)	67 (2.8)	0.01
Proton pump inhibitors	1221 (38.9)	2076 (38.9)	0.00	908 (38.5)	940 (39.8)	−0.03
Others						
Kim et al. [35] CFI, mean (SD)	0.2 (0.1)	0.2 (0.1)	−0.12	0.2 (0.1)	0.2 (0.1)	−0.06
HAS-BLED score, mean (SD)	3.5 (1.3)	3.6 (1.3)	−0.01	3.6 (1.3)	3.6 (1.3)	−0.01
Follow-up time (d)						
Mean (SD)	88.4 (66.0)	92.2 (66.6)	-	89.2 (66.3)	88.8 (67.1)	-
Median (Q1, Q3)	74 (30, 163)	81 (30, 175)	-	74 (30, 163)	75 (30, 170)	-

Unless otherwise specified, baseline characteristics are presented as counts and percentages. Q1 and Q3 represent the lower and upper quartiles, respectively.

CFI, claims-based frailty index; COX-2, cyclooxygenase-2; DOAC, direct oral anticoagulant; DVT, deep vein thrombosis; HAS-BLED, Hypertension, Abnormal renal/liver function, Stroke, Bleeding history or predisposition, Labile international normalized ratio, Elderly (>65 years), Drugs/alcohol concomitantly score; NSAID, nonsteroidal anti-inflammatory drug; PE, pulmonary embolism; PSM, propensity score matching; Std diff., standard difference; VTE, venous thromboembolism.

a A select number of covariates is presented in Table 1; the complete list of covariates is presented in Supplementary Table 5.

After PSM, our analysis cohorts included 2361 DOAC-warfarin pairs (Table 1, Supplementary Table 5), 895 apixaban-warfarin pairs (Supplementary Table 7), 2161 rivaroxaban-warfarin pairs (Supplementary Table 8), and 895 apixaban-rivaroxaban pairs (Supplementary Table 9). The baseline characteristics of the various exposure cohorts were reasonably balanced after PSM, with standard differences of <10% after adjustment (Table 1, Supplementary Tables 5 and 7–9).

### 3.2 Clinical outcomes

#### 3.2.1 Primary outcome

**Figure 2:**
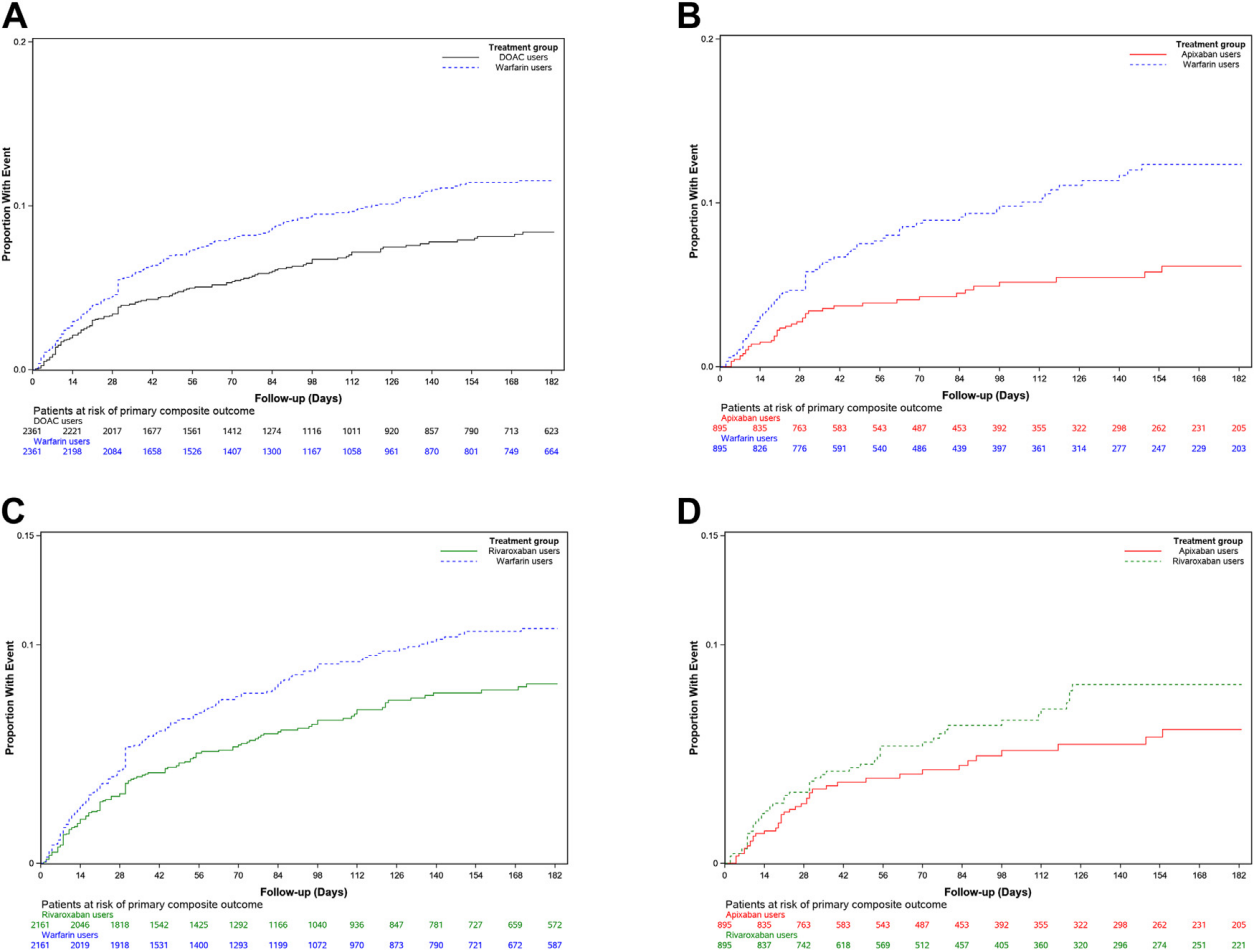
Kaplan–Meier curve for the primary outcome in patients with acute venous thromboembolism and chronic liver disease by treatment groups. (A) Direct oral anticoagulants (DOACs) vs warfarin (top left), (B) apixaban vs warfarin (top right), (C) rivaroxaban vs warfarin (bottom left), and (D) apixaban vs rivaroxaban (bottom right).

After PSM, compared to warfarin, DOACs as a class had reduced risk of the primary outcome—the composite of hospitalization for recurrent VTE and hospitalization for major bleeding (HR, 0.72; 95% CI, 0.61-0.85; Table 2). Similarly, compared with warfarin, individual DOACs, whether apixaban (HR, 0.48; 95% CI, 0.35-0.66) or rivaroxaban (HR, 0.73; 95% CI, 0.61-0.88; Table 3) were associated with reduced risk of the primary outcome. The risk of the primary outcome was similar between apixaban and rivaroxaban (HR, 0.68; 95% CI, 0.43-1.08; Table 3). The cumulative incidence curve for the primary outcome is presented in Figure 2A–D.

**Table 2:** Incidence rate and effect estimates for clinical outcomes in patients with acute venous thromboembolism and chronic liver disease initiating direct oral anticoagulants vs warfarin.

Clinical outcomes	Before PSM				After PSM				Unmatched HR (95% CI)	PSM HR (95% CI)
	DOACs (*n* = 3140; 37%)		Warfarin (*n* = 5337; 63%)		DOACs (*n* = 2361; 50%)		Warfarin (*n* = 2361; 50%)			
	*n*	IR/100 PY	*n*	IR/100 PY	*n*	IR/100 PY	*n*	IR/100 PY		
Primary outcome	176	20.7	479	31.1	146	22.5	209	32.6	0.65 (0.55-0.77)	0.72 (0.61-0.85)
Effectiveness outcomes										
Recurrent VTE	49	5.7	116	7.3	40	6.9	50	8.5	0.75 (0.54-1.05)	0.81 (0.59-1.12)
Death	230	26.7	363	22.8	180	29.1	168	26.8	1.15 (0.98-1.36)	1.09 (0.92-1.30)
Safety outcomes										
Major bleeding	133	15.5	385	24.8	112	16.6	167	25.2	0.62 (0.51-0.75)	0.69 (0.57-0.84)
CRNMB	643	83.4	1314	95.7	503	86.8	635	109.2	0.86 (0.78-0.95)	0.78 (0.71-0.86)
Clinically relevant bleeding	674	87.8	1413	103.7	531	91.9	663	113.9	0.84 (0.78-0.95)	0.79 (0.72-0.87)
Other composite outcomes										
Recurrent VTE or clinically relevant bleeding	706	92.4	1479	109.4	556	97.1	686	119.3	0.83 (0.76-0.91)	0.80 (0.73-0.88)
Any clinical outcome	867	113.9	1722	127.8	685	120.8	786	138.3	0.88 (0.81-0.95)	0.85 (0.78-0.93)

The primary outcome measure was a composite of hospitalization for recurrent venous thromboembolism and hospitalization for major bleeding. Clinically relevant bleeding was a component of the hospitalization for major bleeding and clinically relevant nonmajor bleeding.

CRNMB, clinically relevant nonmajor bleeding; DOAC, direct oral anticoagulant; HR, hazards ratio; IR, incidence rate; PSM, propensity score matching; PY, person-years; VTE, venous thromboembolism.

**Table 3:** Incidence rate and effect estimates for clinical outcomes in patients with acute venous thromboembolism and chronic liver disease initiating a specific direct oral anticoagulant compared to warfarin or another direct oral anticoagulant.

Clinical outcomes	Before PSM				After PSM				Unmatched HR (95% CI)	PSM HR (95% CI)
	Comparator		Reference group		Comparator		Reference group			
	*n*	IR/100 PY	*N*	IR/100 PY	*n*	IR/100 PY	*N*	IR/100 PY		
Primary outcome										
Apixaban vs warfarin	40	17.3	479	31.1	40	16.7	81	37.4	0.54 (0.39-0.74)	0.48 (0.35-0.66)
Rivaroxaban vs warfarin	131	21.9	479	31.1	131	21.3	180	30.8	0.70 (0.57-0.85)	0.73 (0.61-0.88)
Apixaban vs rivaroxaban	40	17.3	131	21.9	40	17.0	54	24.7	0.77 (0.54-1.09)	0.68 (0.43-1.08)
Effectiveness outcomes										
Recurrent VTE										
Apixaban vs warfarin	12	5.1	116	7.3	12	4.8	25	12.8	0.66 (0.36-1.20)	0.47 (0.26-0.86)
Rivaroxaban vs warfarin	34	5.6	116	7.3	34	6.2	46	8.2	0.75 (0.51-1.10)	0.81 (0.57-1.14)
Apixaban vs rivaroxaban	12	5.1	34	5.6	12	5.3	17	7.4	0.88 (0.46-1.71)	0.92 (0.42-2.02)
All-cause mortality										
Apixaban vs warfarin	88	37.9	363	22.8	86	37.3	96	41.1	1.56 (1.24-1.98)	1.11 (0.87-1.41)
Rivaroxaban vs warfarin	141	23.2	363	22.8	140	23.7	142	23.6	1.0 (0.83-1.22)	1.05 (0.87-1.27)
Apixaban vs rivaroxaban	88	37.9	141	23.2	86	40.4	89	41.9	1.56 (1.20-2.05)	1.18 (0.81-1.71)
Safety outcomes										
Major bleeding										
Apixaban vs warfarin	29	12.6	385	24.8	29	11.8	61	27.2	0.49 (0.33-0.71)	0.43 (0.30-0.63)
Rivaroxaban vs warfarin	102	16.9	385	24.8	102	15.9	142	23.4	0.68 (0.54-0.84)	0.72 (0.58-0.89)
Apixaban vs rivaroxaban	29	12.6	102	16.9	29	11.7	41	18.3	0.72 (0.48-1.08)	0.60 (0.35-1.06)
CRNMB										
Apixaban vs warfarin	161	75.6	1314	95.7	161	73.4	250	119.5	0.77 (0.65-0.91)	0.61 (0.52-0.72)
Rivaroxaban vs warfarin	461	85.8	1314	95.7	461	85.7	554	103.6	0.89 (0.80-0.99)	0.83 (0.75-0.92)
Apixaban vs rivaroxaban	161	75.6	461	85.8	161	73.6	199	97.6	0.87 (0.72-1.04)	0.74 (0.58-0.94)
Clinically relevant bleeding										
Apixaban vs warfarin	168	79.2	1413	103.7	168	77.4	255	121.9	0.75 (0.64-0.88)	0.61 (0.52-0.72)
Rivaroxaban vs warfarin	483	90.1	1413	103.7	483	89.9	578	108.6	0.86 (0.78-0.96)	0.83 (0.75-0.92)
Apixaban vs rivaroxaban	168	79.2	483	90.1	168	77.9	209	102.4	0.86 (0.72-1.03)	0.73 (0.58-0.93)
Other composite outcomes										
Recurrent VTE or clinically relevant bleeding										
Apixaban vs warfarin	175	82.5	1479	109.4	175	80.6	264	129.6	0.74 (0.63-0.86)	0.62 (0.53-0.73)
Rivaroxaban vs warfarin	506	95.1	1479	109.4	506	94.9	600	113.6	0.86 (0.78-0.95)	0.83 (0.75-0.91)
Apixaban vs rivaroxaban	175	82.5	506	95.1	175	81.4	219	108.5	0.86 (0.72-1.02)	0.74 (0.59-0.94)
Any clinical outcome										
Apixaban vs warfarin	234	111.4	1722	127.8	234	107.5	320	158.0	0.85 (0.74-0.98)	0.71 (0.61-0.82)
Rivaroxaban vs warfarin	605	113.9	1722	127.8	605	114.0	686	130.5	0.89 (0.81-0.97)	0.86 (0.78-0.94)
Apixaban vs rivaroxaban	234	111.4	605	113.9	234	112.9	283	142.7	0.97 (0.84-1.13)	0.83 (0.67-1.01)

The primary outcome measure was a composite of hospitalization for recurrent venous thromboembolism and hospitalization for major bleeding. Clinically relevant bleeding was a component of the hospitalization for major bleeding and clinically relevant nonmajor bleeding.

CRNMB, clinically relevant nonmajor bleeding; HR, hazards ratio; IR, incidence rate; PSM, propensity score matching; PY, person-years; VTE, venous thromboembolism.

#### 3.2.2 Bleeding risks

Significantly lower risks of hospitalization for major bleeding were observed with DOACs as a class (HR, 0.69; 95% CI, 0.57-0.84; Table 2), apixaban (HR, 0.43; 95% CI, 0.30-0.63) or rivaroxaban (HR, 0.72; 95% CI, 0.58-0.89) vs warfarin but not apixaban vs rivaroxaban (HR, 0.60; 95% CI, 0.35-1.06) (Tables 2 and 3, Supplementary Figure 2A–D).

#### 3.2.3 Recurrent VTE

The risk of hospitalization for recurrent VTE was similar with DOACs as a class (HR, 0.81; 95% CI, 0.59-1.12) and rivaroxaban (HR, 0.81; 95% CI, 0.57-1.14) compared with warfarin and apixaban vs rivaroxaban (HR, 0.92; 95% CI, 0.42-2.02). Conversely, compared with warfarin, apixaban was associated with a significantly reduced risk of hospitalization for recurrent VTE (HR, 0.47; 95% CI, 0.26-0.86) (Tables 2 and 3, Supplementary Figure 3A–D).

#### 3.2.4 Mortality risks

No difference was observed in risk of all-cause mortality for exposure to any or specific DOACs vs warfarin or apixaban vs rivaroxaban (Tables 2 and 3). Although with wide CIs, the IRs and upper limit of the effect estimate for all-cause mortality were consistently higher with DOACs as a class vs warfarin (1.30), apixaban (1.41), or rivaroxaban (1.27) compared with warfarin, and apixaban vs rivaroxaban (1.71), suggesting the possibility of a nonnull effect.

### 3.3 Secondary and subgroup analyses

Similar to the primary findings, compared to warfarin, lower risk of the composite outcome was seen in individuals with cirrhosis (compensated or decompensated), and 36% reduction in risk of the primary outcome was observed with DOACs vs warfarin (HR, 0.64; 95% CI, 0.43-0.96), specifically a 56% reduced risk in the subset of subjects with decompensated cirrhosis (HR, 0.44; 95% CI, 0.26-0.73) (Table 4). The findings from subgroup analyses suggest that the magnitude of association between DOACs and warfarin was moderated by the presence of cancer, chronic renal disease, and NAFLD/nonalcoholic steatohepatitis (Table 4). These analyses should be interpreted in context of a relatively small sample size.

**Table 4:** Secondary and subgroup analyses for the primary composite outcome in patients with acute venous thromboembolism and chronic liver disease initiating direct oral anticoagulants compared with warfarin.

Analysis	Before PSM				After PSM				Unmatched HR (95% CI)	PSM HR (95% CI)
	DOACs		Warfarin		DOACs		Warfarin			
	*n*	IR/100 PY	*n*	IR/100 PY	*n*	IR/100 PY	*n*	IR/100 PY		
Secondary analyses										
Advanced liver disease										
Cirrhosis^a^	62	31.5	199	47.3	55	33.3	87	52.6	0.65 (0.49-0.87)	0.64 (0.43-0.96)
Decompensated cirrhosis only	32	27.1	131	54.1	32	27.3	69	62.8	0.50 (0.34-0.74)	0.44 (0.26-0.73)
Subgroup analyses										
Age categories										
≥65 y	90	22.0	263	33.4	90	21.4	145	36.7	0.64 (0.51-0.82)	0.83 (0.57-1.22)
<65 y	86	19.1	216	28.6	62	22.6	77	26.2	0.67 (0.52-0.86)	0.74 (0.49-1.11)
Sex										
Female	97	22.5	262	32.9	77	23.3	128	39.5	0.67 (0.53-0.84)	0.56 (0.40-0.79)
Male	79	18.8	217	29.1	62	20.4	84	26.8	0.63 (0.49-0.82)	0.80 (0.55-1.19)
Cancer										
Yes	116	28.2	290	39.4	91	31.8	114	38.5	0.71 (0.57-0.88)	0.75 (0.53-1.05)
No	60	13.6	189	23.4	56	16.5	88	27.3	0.56 (0.42-0.75)	0.58 (0.39-0.86)
Chronic renal disease										
Yes	40	28.4	94	32.2	31	30.4	42	36.4	0.84 (0.58-1.22)	0.80 (0.46-1.41)
No	136	19.2	385	30.8	100	18.3	165	31.9	0.62 (0.50-0.74)	0.54 (0.39-0.73)
Diabetes										
Yes	66	23.7	179	32.7	44	19.8	77	36.3	0.71 (0.53-0.94)	0.46 (0.29-0.73)
No	110	19.2	300	30.2	79	17.1	135	32.0	0.62 (0.50-0.78)	0.57 (0.40-0.81)
NAFLD/NASH only										
Yes	40	15.2	80	19.7	22	12.5	32	18.5	0.75 (0.51-1.09)	0.72 (0.39-1.32)
No	136	23.2	399	35.1	113	23.4	182	37.8	0.65 (0.53-0.78)	0.63 (0.48-0.83)
Provoked VTE										
Yes	133	22.9	404	33.5	109	24.5	160	37.1	0.66 (0.55-0.81)	0.73 (0.55-0.98)
No	43	15.9	75	22.4	32	19.4	42	22.2	1.70 (0.48-1.02)	0.72 (0.41-1.26)

DOAC, direct oral anticoagulant; HR, hazards ratio; IR, incidence rate; NAFLD, nonalcoholic fatty liver disease; NASH, nonalcoholic steatohepatitis; PSM, propensity score matching; PY, person-years; VTE, venous thromboembolism.

a Cirrhosis is a composite of compensated and decompensated cirrhosis. The primary outcome measure was a composite of hospitalization for recurrent venous thromboembolism and hospitalization for major bleeding.

### 3.4 Sensitivity analyses

Our primary conclusions for DOACs vs warfarin remained largely consistent across sensitivity analyses, including using an alternative follow-up period of up to 3 months (Supplementary Table 10) or 12 months (Supplementary Table 11), an intent-to-treat design (Supplementary Table 12), reducing the maximum gap between refills from 30 days in the primary analysis to 7 or 14 days and based on half-life of each oral anticoagulant (Supplementary Table 13), and cohort restricted to subjects that initiated treatment prior to June 30, 2017 (Supplementary Table 14). There was no meaningful difference between the overall conclusions from the primary models comparing DOACs as a class vs warfarin and alternative Fine and Gray subdistribution hazard models with death treated as a competing risk; for DOACs as a class vs warfarin, the subdistribution HR for the primary outcome was 0.73 (95% CI, 0.61, 0.88), subdistribution HR for hospitalization for recurrent VTE was 0.96 (95% CI, 0.68, 1.38), and subdistribution HR for hospitalization for major bleeding was 0.67 (95% CI, 0.54, 0.82).

Further, we observed an E-value of 2.12 and lower bound of 1.63 for the primary outcome analysis comparing DOACs with warfarin. This implies that the observed effect estimate for the primary outcome could be explained away by a confounder unmeasured in our analyses, uncorrelated with measured confounders, but associated with both the treatment and outcome by a risk ratio of ≥1.63. In the falsification analysis with pneumonia as the outcome, the PSM HR for DOACs vs warfarin was 0.96 (95% CI, 0.77-1.19) and 0.80 (95% CI, 0.74-0.88) for hd-PSM analyses, thus suggesting the absence of substantial residual confounding.

## 4 Discussion

To the best of our knowledge, this is the first large population-based, comparative effectiveness and safety study of oral anticoagulants in patients with VTE and chronic liver disease. The current study adds 3 salient findings to the evidence needed to guide the selection of oral anticoagulation therapy in patients with VTE and chronic liver disease in contemporary clinical settings.

First, among patients with VTE and chronic liver disease, DOACs (as a class) had a more favorable benefit-risk profile than that of warfarin. Of note, the oral direct FXa inhibitors comprised 97% of the 3140 DOAC users. Although little data exist on VTE among patients with chronic liver disease [8,11], this finding is consistent with evidence from several studies of different designs suggesting that DOACs are as safe and effective as warfarin, if not superior in individuals with VTE [5,7,[12], [13], [14], [15],^46^]. The larger net clinical benefit observed with DOACs compared to that with warfarin may be partly explained by its more stable pharmacokinetic characteristics, no requirement for international normalized ratio monitoring, and fewer food and drug interactions. More so, human cytochrome-450 enzymes are markedly altered in chronic liver disease, particularly cirrhosis [8,11]. While warfarin is primarily metabolized by cytochrome enzymes and relies on the liver for clearance, DOACs are generally less reliant on these enzymes for metabolism [8,11].

Second, the magnitude of net clinical benefit differed between individual oral direct FXa inhibitors compared to warfarin and between oral direct FXa inhibitors. Overall, compared to warfarin, the magnitude of clinical benefit was consistently larger for apixaban than for rivaroxaban and in head-to-head comparison of apixaban and rivaroxaban. Similar findings of lower bleeding risks associated with apixaban vs rivaroxaban, when compared to warfarin, were observed in randomized controlled trials involving patients with atrial fibrillation [47,48] or VTE [[12], [13], [14], [15]] and real-world studies, including head-to-head comparisons of apixaban and rivaroxaban [23,[49], [50], [51], [52]]. For example, in the AMPLIFY acute VTE trial [12], subjects on apixaban vs warfarin had significantly lower risk of major bleeding (HR, 0.31; 95% CI, 0.17-0.55) and clinically relevant bleeding (HR, 0.44; 95% CI, 0.36-0.55). However, in the EINSTEIN-DVT trial [13], the risk of major bleeding (HR, 0.65; 95% CI, 0.33-1.30) or clinically relevant bleeding (HR, 0.97; 95% CI, 0.76-1.22) did not significantly differ between rivaroxaban vs enoxaparin and warfarin or acenocoumarol. Although causal mechanisms are still unclear, it has been hypothesized that the lower bleeding risks observed with apixaban are attributable in part to lower peak levels from twice-daily dosing of apixaban vs the once-daily dosing regimen of rivaroxaban [53].

Third, our results suggest that DOACs may serve as an alternative to warfarin in individuals with VTE and cirrhosis, including decompensated cirrhosis. Importantly, these findings address a question yet to be answered by clinical trials while extending available real-world data, albeit from small sample size studies [16,[54], [55], [56]], on the use of DOACs in this most at-risk population. Future studies using prospectively collected data, involving a larger sample size, and further delineating which DOAC and what dosage of DOAC is associated with optimal clinical benefit in individuals with VTE and advanced liver disease are, however, needed.

The annualized risk for major bleeding in the VTE clinical trials for DOACs ranged between 1.2% and 2.2% [57], with an incidence of 0.6% and 0.5% in patients randomized to apixaban or rivaroxaban, respectively [12,13]. Conversely, 3.3% (11.8 IR/person-year) and 4.7% (15.9 IR/person-year) of patients on apixaban and rivaroxaban treatment, respectively, in the current study had medical encounters indicative of hospitalization for major bleeding. Despite the well-known benefits of oral anticoagulation treatment in reducing thrombotic risk, the finding of more than 5-fold the risk of major bleeding events in the current study compared to the VTE clinical trials and other real-world studies involving VTE [5,7,[12], [13], [14], [15],^46^] highlight the need for careful consideration of risks vs benefit of oral anticoagulation in these most at-risk patients.

### 4.1 Limitations

First, considering the retrospective nature of the data, our study is prone to several forms of measurement errors, including exposure and outcome misclassification. Nonetheless, the consistent findings across several sensitivity analyses suggest the robustness of our primary analyses. Second, although we carefully adjusted for confounding by several well-known confounders, this study may still be subject to residual and unmeasured confounding. For example, unmeasured confounding may have occurred due to lack of information on over-the-counter use of aspirin and nonsteroidal anti-inflammatory drugs, drugs that are well-known to increase bleeding risks. The findings from negative control analysis and hd-PSM, however, provide some reassurance of the absence of substantial residual confounding.

Third, our data lacked information to determine the time in the therapeutic range. Further, clinical and laboratory data needed to calculate Child–Pugh scores for assessing chronic liver disease severity were either missing or grossly underreported, thus precluding our ability to calculate Child–Pugh scores or use other predictive models (eg, the Model for End-Stage Liver Disease score) that rely on laboratory data. This issue of lack of laboratory data, while mitigated by our use of validated algorithms for cirrhosis, has been duly reported by other investigators utilizing claims databases for postmarket safety studies involving claims data [10,58]. Fourth, our data lack information on race and sociodemographic variables, factors that may moderate burden of disease and access to medical services. Lastly, the present study involved subjects with private health insurance in the United States who may have lower burden of underlying comorbidities and higher access to healthcare services compared to unemployed or uninsured patients. Since the biologic effects of oral anticoagulants are unexpected to change by insurance status, our overall findings may be generalizable to the general population of individuals with VTE and chronic liver disease. Despite these limitations, given the current lack of data from randomized controlled trials, evidence to guide oral anticoagulant treatment decision-making in this at-risk population will need to accrue in the meantime from real-world clinical settings.

## 5 Conclusion

Among subjects with VTE and chronic liver disease receiving oral anticoagulants, DOACs were associated with significantly lower risk of a composite of hospitalization for recurrent VTE and hospitalization for major bleeding compared to warfarin. The observed differences favoring DOACs were greater with apixaban than with rivaroxaban. The relative differences, favoring DOACs over warfarin, remained consistent among individuals with advanced chronic liver disease. These data suggest that DOACs may be a suitable alternative to warfarin in this setting. Patients with chronic liver disease should be included in future randomized trials to confirm these findings.
