# Supplementary material for: Venous thromboembolism, chronic liver disease and anticoagulant choice: effectiveness and safety of direct oral anticoagulants versus warfarin
Source: Res Pract Thromb Haemost. 2023 Dec 9;8(1):102293. doi: 10.1016/j.rpth.2023.102293 (PMC10805675; doi:10.1016/j.rpth.2023.102293)
Supplement: Supplementary Material [file mmc1.docx]

**Venous thromboembolism, chronic liver disease and anticoagulant choice: effectiveness and safety of direct oral anticoagulants versus warfarin**

**Authors:** Oluwadolapo D. Lawal, PhD, MPH; ^1^ Herbert D. Aronow, MD;^2,3^ Anne Hume, PharmD;^1^ Fisayomi Shobayo, MD;^4^ Kelly Matson, PharmD;^1^ Marilyn Barbour, PharmD; ^1^ Yichi Zhang, PhD;^5^ Xuerong Wen, PhD, MPH^1^

**Affiliations:**

^1^ Department of Pharmacy Practice, College of Pharmacy, University of Rhode Island, Kingston, Rhode Island, United States.

^2^ Lifespan Cardiovascular Institute, Providence, Rhode Island, United States

^3^ Warren Alpert Medical School of Brown University, Providence, Rhode Island, United States

^4^ Department of Cardiology, University of Texas Health Science Center, Houston, Texas, United States.

^5^ Department of Computer Sciences and Statistics, University of Rhode Island, Kingston, Rhode Island, United States

**Address correspondence to:**

Xuerong Wen, Associate Professor of Pharmacoepidemiology and Health Outcomes

University of Rhode Island, College of Pharmacy, Department of Pharmacy Practice

7 Greenhouse Road, Suite 265F, Kingston, RI, USA 02881

Email: [xuerongwen@uri.edu](mailto:xuerongwen@uri.edu); Tel: 401-874-4547

**Appendix methods**

**Appendix Figures**

1. Appendix Figure 1. Study design
2. Appendix Figure 2. Kaplan Meier curve for major bleeding in patients with acute venous thromboembolism and chronic liver disease by treatment groups
3. Appendix Figure 3. Kaplan Meier curve for recurrent venous thromboembolism in patients with acute venous thromboembolism and chronic liver disease by treatment groups

**Appendix Tables**

1. Appendix Table 1. Algorithms and corresponding International Classification of Disease, 9^th^ and 10^th^ edition, Clinical Modification (ICD-9-CM or ICD-10-CM) codes for identifying the study cohort.
2. Appendix 2. Algorithms and corresponding International Classification of Disease, 9^th^ and 10^th^ edition, Clinical Modification (ICD-9-CM or ICD-10-CM) codes for identifying clinical outcomes.
3. Appendix Table 3: Adjusted covariates and time of assessment
4. Appendix Table 4: Distribution of treatment groups in patients with acute venous thromboembolism and chronic liver disease by index dose, treatment discontinuation and switching.
5. Appendix Table 5. Baseline characteristics of patients with acute venous thromboembolism and chronic liver disease initiating DOACs versus warfarin before and after propensity score matching
6. Appendix Table 6: Distribution of the types of chronic liver disease among patients with acute venous thromboembolism and chronic liver disease
7. Appendix Table 7: Baseline characteristics of patients with acute venous thromboembolism and chronic liver disease initiating apixaban versus warfarin before and after propensity score matching.
8. Appendix Table 8: Baseline characteristics of patients with acute venous thromboembolism and chronic liver disease initiating rivaroxaban versus warfarin before and after propensity score matching.
9. Appendix Table 9: Baseline characteristics of patients with acute venous thromboembolism and chronic liver disease initiating apixaban versus rivaroxaban before and after propensity score matching.
10. Appendix Table 10: Incidence rates and effect estimates for clinical outcomes by treatment groups using an alternative three month follow-up period.
11. Appendix Table 11: Incidence rates and effect estimates for clinical outcomes by treatment groups using an alternative twelve month follow-up period.
12. Appendix Table 12: Incidence rates and effect estimates for clinical outcomes by treatment groups using an intent-to-treat design.
13. Appendix Table 13: Incidence rates and effect estimates for clinical outcomes by treatment groups after varying the maximum gap between prescription refills.
14. Appendix Table 14: Incidence rates and effect estimates for clinical outcomes in cohort restricted to patients that initiated oral anticoagulation between January 1, 2011, and June 30, 2017

**APPENDIX METHODS**

**Allowable gap between prescriptions.** A 30-day gap was allowed between refills to reflect real-world settings wherein medication adherence is often <100%, or for other reasons such as patients inadvertently not refilling medications on time. However, the half-life of all DOACs and warfarin are considerably <30 days; 20 – 60 hours for warfarin, approximately 12 hours for apixaban, 7 – 13 hours for rivaroxaban, 12 – 17 hours for dabigatran, and 10 – 14 hours for edoxaban.^1,2^ Therefore, we performed sensitivity analyses around the allowable gap to increase the likelihood that patients remained anticoagulated during follow-up, Specifically, we used more conservative gaps of (a) a 7-day gap; (b) 14 days between prescriptions; and lastly (c) a gap based on half-life of respective eligible drugs, operationalized as a gap of 7-day for warfarin users and 3-day for DOACs.

**Liver severity**. Chronic liver disease severity is often determined using summed scores from the Child-Pugh classification. However, limited (or lack of) availability of the clinical and laboratory components of the Child-Pugh score precluded our ability to effectively compute Child-Pugh scores for included patients. Ascites and encephalopathy - the clinical components of the Child-Pugh score - are unavailable in our data. Although CPT codes for the laboratory components of the Child-Pugh score - bilirubin, albumin, and prothrombin time or international normalized ratio (INR) - were present in 7141 (84%) patients, results from these tests were available in only a subset of patients. Specifically, 1853 (22%) patients had ≥ 1 testing results, 610 (7.2%) DOAC-exposed had testing results for bilirubin and albumin, while only 121 (1.4%) warfarin-exposed patients had results for bilirubin, albumin and prothrombin time or INR. Collectively, the findings of a relatively large number of patients with CPT scores but limited laboratory results suggests that these serologic tests were indeed frequently performed but underreported.

Given this limitation, we alternatively identified patients with cirrhosis and its more advanced form decompensated cirrhosis using validated algorithms based on diagnosis codes. ^3–5^ Individuals with cirrhosis included those with diagnosis claims indicative of compensated or decompensated cirrhosis. Among individuals with cirrhosis, hepatic decompensation was defined by the presence of ≥ 1 inpatient or ≥ 2 outpatient claims for any of the following complications of cirrhosis during the baseline period: ascites, variceal hemorrhage, hepatorenal syndrome, hepatic encephalopathy, hepatocellular carcinoma and portal hypertension (**Appendix Table 1)** ^3–5^

**Appendix Figure 1. Study design**

**
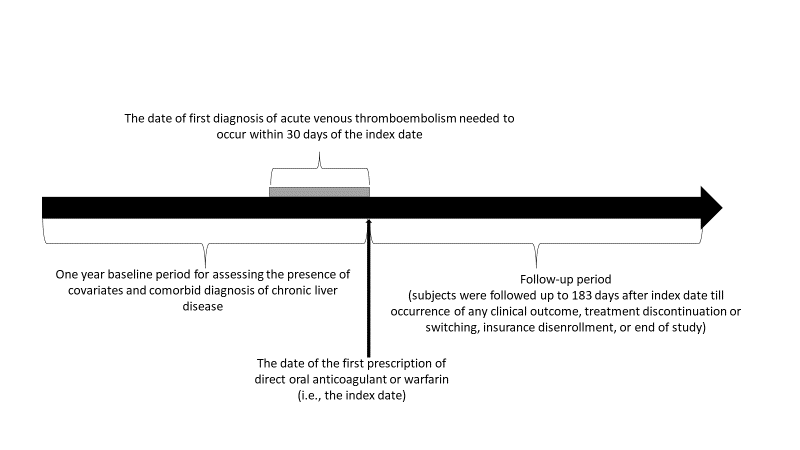
**

**Appendix Figure 2****. Kaplan Meier curve for major bleeding in patients with acute venous thromboembolism and chronic liver disease by treatment groups**


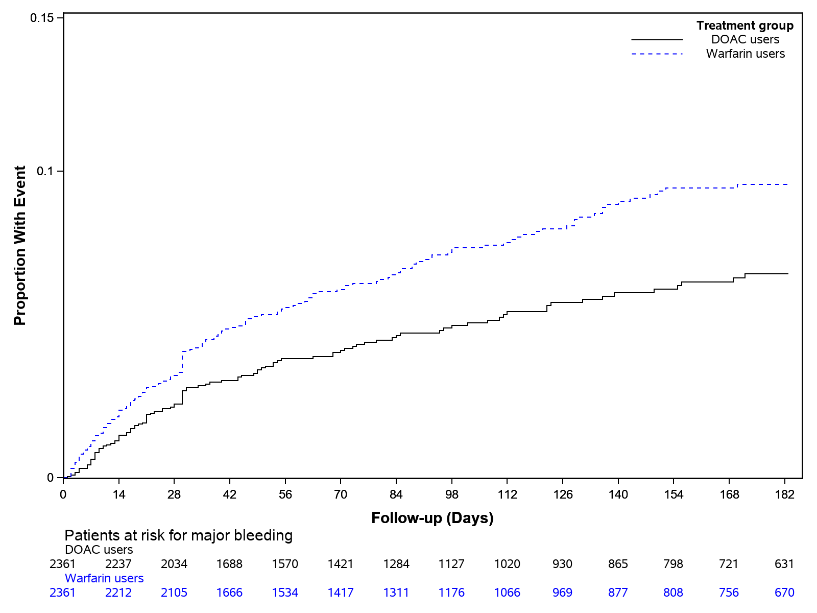

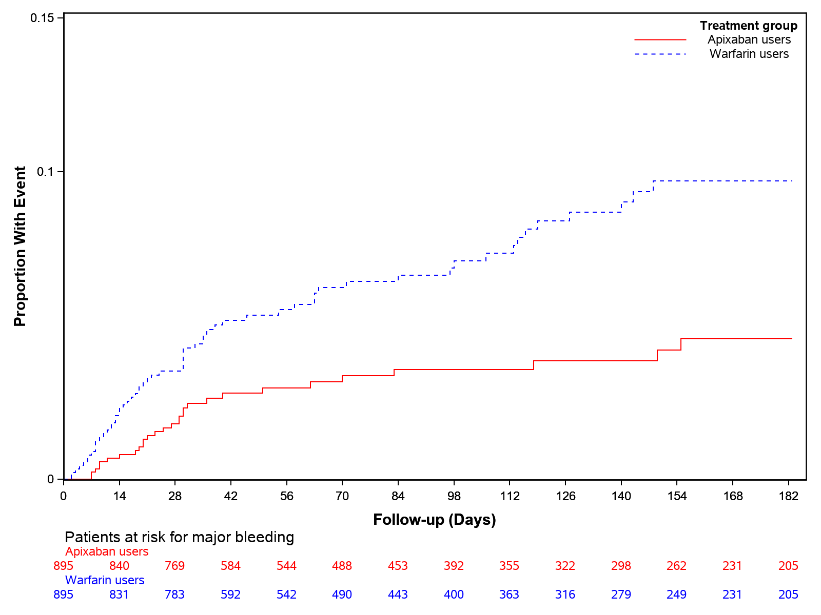


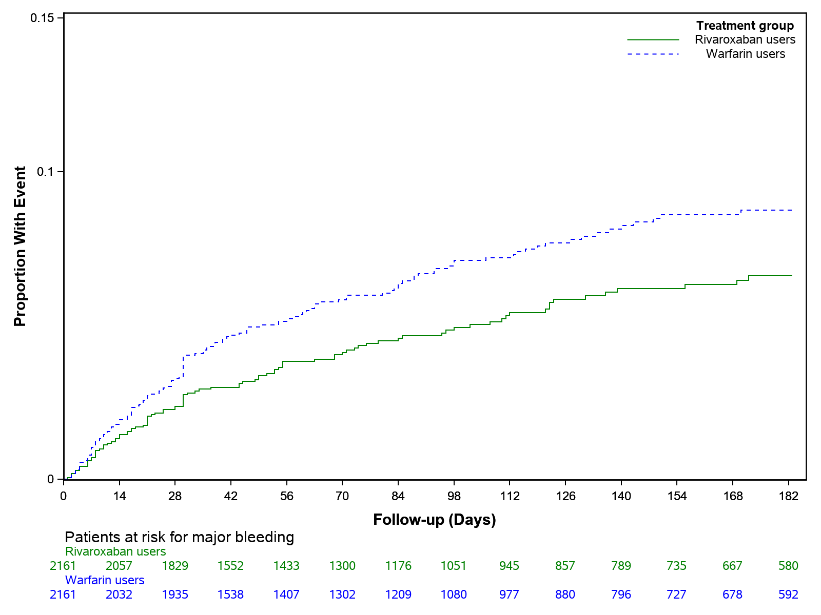

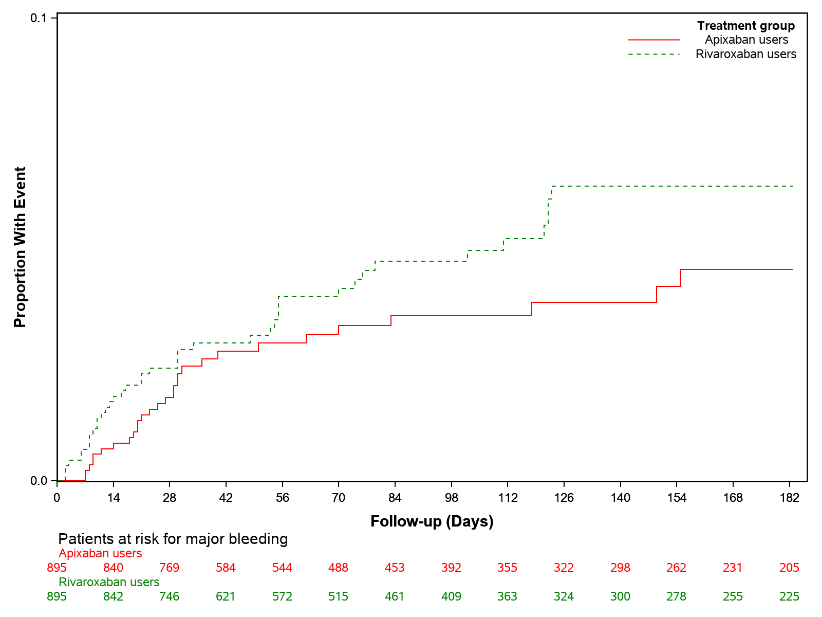


Abbreviations. DOACs, direct oral anticoagulants. DOACs vs warfarin (Figure 2a; top left), apixaban vs warfarin (Figure 2b; top right), rivaroxaban vs warfarin (Figure 2c; bottom left); apixaban vs rivaroxaban (Figure 2d; bottom right).

**Appendix Figure 3. Kaplan Meier curve for recurrent venous thromboembolism in patients with acute venous thromboembolism and chronic liver disease by treatment groups**


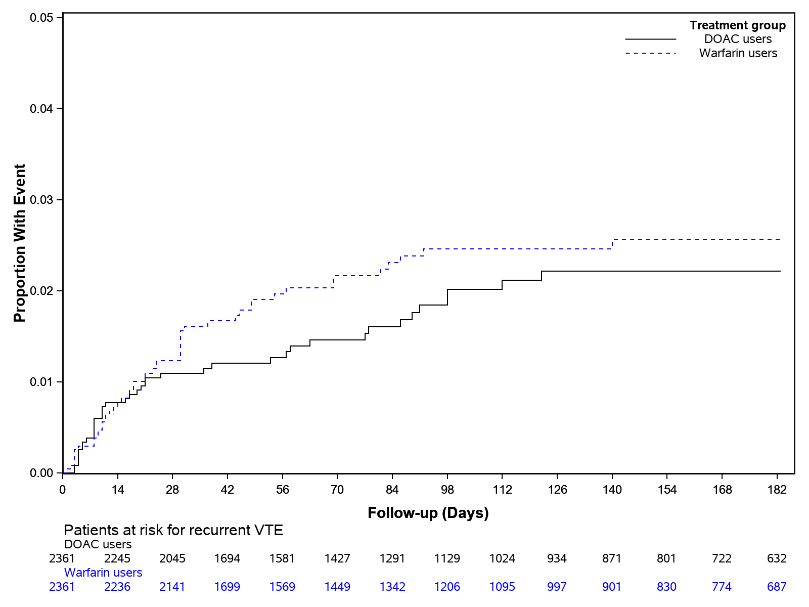

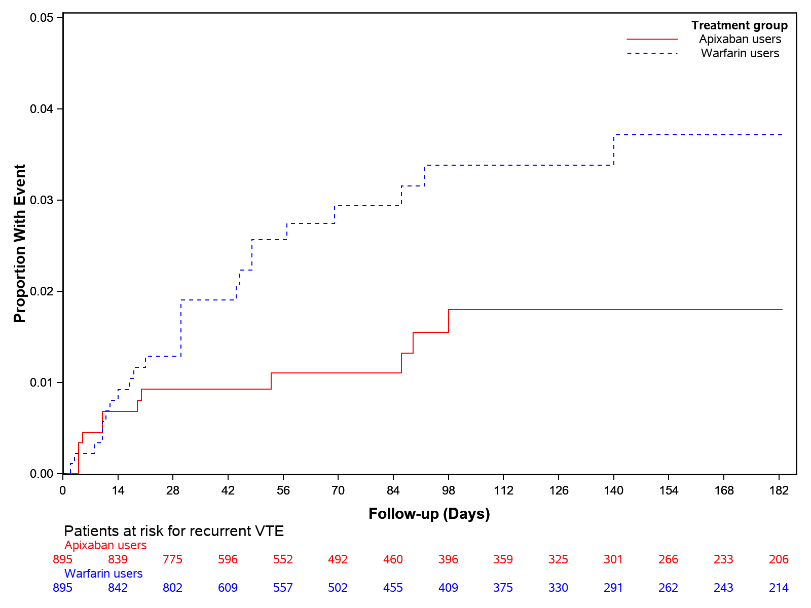


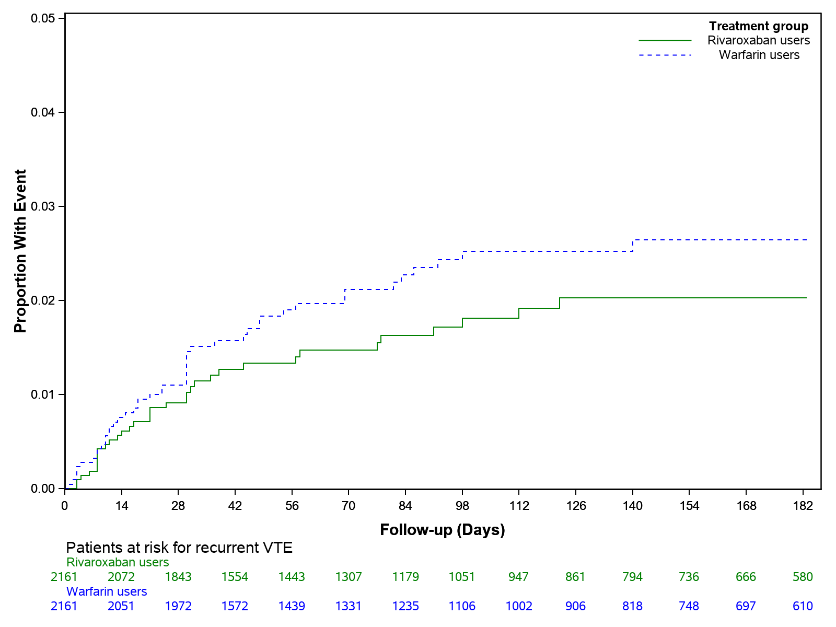

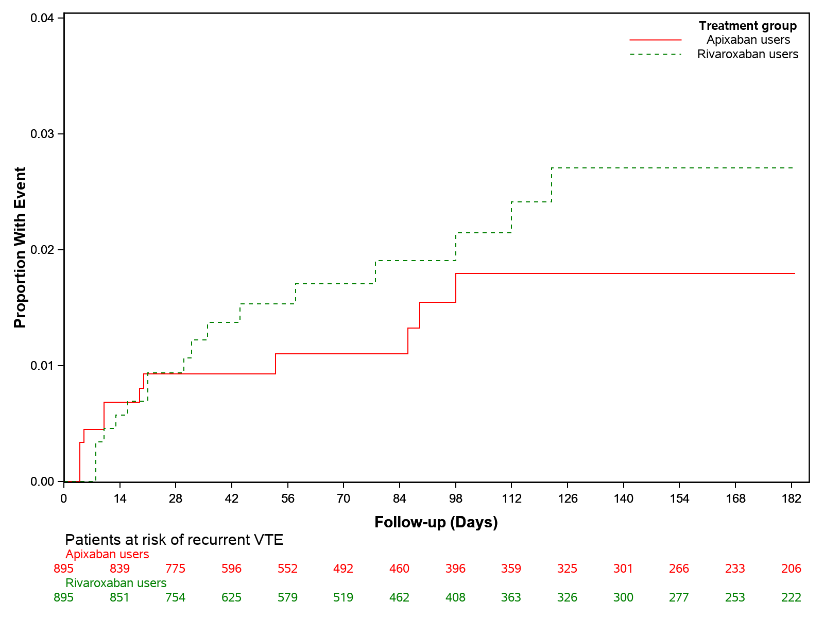


Abbreviations. DOACs, direct oral anticoagulants. DOACs vs warfarin (Figure 3a; top left), apixaban vs warfarin (Figure 3b; top right), rivaroxaban vs warfarin (Figure 3c; bottom left); apixaban vs rivaroxaban (Figure 3d; bottom right).

**Appendix Table 1. Algorithms and corresponding International Classification of Disease, 9^th^ and 10^th^ edition, Clinical Modification (ICD-9-CM or ICD-10-CM) codes for identifying the study cohort.**

| **Clinical event** | **ICD-9 diagnosis codes** | **ICD-10 diagnosis codes** | **Additional information** | **References** |
| --- | --- | --- | --- | --- |
| Acute VTE | 415.11, 415.12, 415.13, 415.19, 451.11, 451.19, 451.2, 451.81, 451.82, 451.83, 451.84, 451.89, 451.9, 453.40, 453.41, 453.42, 453.81, 453.82, 453.83, 453.84, 453.85, 453.86, 453.87, 453.89, 453.9 | I26.9, I26.90, I26.92, I26.99, I26.0, I26.01, I26.02, I26.09, I80.2, I80.3, I80.1, I82.4, I82.8, I80.9, I82.9, I82.90, I80.8, O22.3, O22.9, O87.1 | VTE was defined as ≥ 1 diagnosis in primary or secondary position present in inpatient or outpatient claims for VTE. Further, patients were required to fill an oral anticoagulant within 30 days of hospital discharge (if in-patient) or outpatient service date for the first VTE diagnosis. | ^6–8^ |
| **Common etiologies of chronic liver disease** | | | | ^3–5, 9,10^ |
| Cirrhosis | 155.0, 155.2, 456.0 - 456.2, 571.2, 571.5, 572.2, 572.3, 572.4, 789.5 | I85.9, I98.2, I86.4, I85.0, I98.3, R18.xx, K76.7, K76.6, C22.0 | Chronic liver disease was identified by the presence of ≥ 1 inpatient or ≥ 2 outpatient claims for non-idiopathic conditions associated with prolonged or complete deterioration of liver function. Specific types of liver disease were identified using previous literature, and categories listed in an expert panel consensus statement on NAFLD by the American Association for the Study of Liver disease (AASLD).^9,10^  Among individuals with cirrhosis,^3–5^ we identified patients with decompensated cirrhosis, defined as the presence of ≥ 1 inpatient or ≥ 2 outpatient claims for esophageal varices with bleeding (456.0, 456.20, I85.0, I98.3); ascites (789.5, R18); hepatic encephalopathy (572.2, or diagnosis code for cirrhosis and prescription of lactulose or rifaximin within 3 months of the index date); hepatorenal syndrome (572.4, K76.7); portal hypertension (572.3, K76.6), and hepatocellular carcinoma (155.0, 155.2, C22.0). This algorithm has been reported with PPV between 78% - 91%.^3–5^  In sensitivity analyses, we compared the incidence of clinical outcomes between DOAC and warfarin users. To establish a cohort of individuals with only NAFLD, we followed a hierarchical method reported by Allen et al^10^ and in a consensus statement from the AASLD for identifying NAFLD in administrative claims database^9^ wherein patients with other known causes of chronic liver disease (e.g., cirrhosis, ALD) or diagnosis of alcohol use disorders during the baseline period are systematically excluded. In a previous study,^10^ this algorithm was found to be 85% accurate in identifying true NAFLD cases compared to chart review (i.e., information available in outpatient and hospital notes, images, laboratory results, and histologic data |  |
| Alcoholic liver disease (ALD) | 571.0 – 571.3 | K70.xx |  |  |
| Viral hepatitis | 070.xx | B16.xx – B19.xx |  |  |
| Non-alcoholic liver disease (NAFLD) | 571.8 | K76.0 |  |  |
| Liver disease of genetic causes | 275.0, 277.6, and 275.5 | E83.1, E88.01, E88.0B, E83.01 |  |  |
| Liver diseases of autoimmune causes | 571.6, 576.1 | K83.0A, K83.0F, K74.3, K75.4 |  |  |
| Liver cancer | 155.0, 155.2 | C22.0, C22.9 |  |  |
| Liver failure | 572.8, 570 | K72.1, K72.0, K72.9, I81.9, K75.1, K74.0, K74.1, K74.2 |  |  |
| Liver transplant | V427 | Z94.4 |  |  |
| Budd-Chiari disease | 453.0 | I82.0, K76.5 |  |  |
| Others |  |  |  |  |
| Unspecified chronic hepatitis | 571.4 | K73.9, K73.2, K73.8 |  |  |
| Secondary or unspecified biliary cirrhosis | 571.6 | K74.4, K74.5 |  |  |
| Hepatic fibrosis or sclerosis or fibrosis with sclerosis | 571.9 | K74.0, K74.1, K74.2 |  |  |

Abbreviations. ALD, alcoholic liver disease; AASLD, American Association for the Study of Liver disease; DOAC, direct oral anticoagulants; NAFLD, Non-alcoholic liver disease; PPV, positive predictive value; VTE, venous thromboembolism.

**Appendix 2. Algorithms and corresponding International Classification of Disease, 9^th^ and 10^th^ edition, Clinical Modification (ICD-9-CM or ICD-10-CM) codes for identifying clinical outcomes.**

| **Clinical outcome** | **ICD-9** | **ICD-10** | **Additional information** | **PPV** | **References** |
| --- | --- | --- | --- | --- | --- |
| Acute venous thrombo-embolism  (VTE) | ICD-9-CM: 415.11, 415.12, 415.13, 415.19, 451.11, 451.19, 451.2, 451.81, 451.82, 451.83, 451.84, 451.89, 451.9, 45340, 45341, 45342, 453.81, 453.82, 453.83, 453.84, 453.85, 453.86, 453.87, 453.89, 453.9 | ICD-10-CM: I26.9, I26.90, I26.92, I26.99, I26.0, I26.01, I26.02, I26.09, I80.2, I80.20, I80.201, I80.202, I80.203, I80.209, I80.21, I80.211, I80.212, I80.213, I80.219, I80.222, I80.223, I80.229, I80.23, I80.231, I80.232, I80.233, I80.239, I80.29, I80.291, I80.292, I80.293, I80.299, I80.3, I80.1, I80.10, I80.11, I80.12, I80.13, I82.4, I82.890, I82.89, I82.8, I80.9, I82.9, I82.90, I80.8, O22.3, O22.30, O22.31, O22.32, O22.33, O22.9, O22.90, O22.91, O22.92, O22.93, O87.1 | Recurrent VTE was defined by ≥ 1 inpatient claim with diagnosis codes in the primary position for VTE > 7 days after index VTE event | > 90% | ^6–8^ |
| Bleeding | Gastrointestinal bleeding (GIB)  ICD-9-CM: 456.0, 456.20, 530.21, 530.7, 530.82, 531.00, 531.01, 531.20, 531.21, 531.40, 531.41, 531.60, 531.61, 532.00, 532.01, 532.20, 532.21, 532.40, 532.41, 532.60, 532.61, 533.00, 533.01, 533.20, 533.21, 533.40, 533.41, 533.60, 533.61, 534.00, 534.01, 534.20, 534.21, 534.40, 534.41, 534.60, 534.61, 535.01, 535.11, 535.21, 535.31, 535.41, 535.51, 535.61, 537.83, 537.84, 562.02, 562.03, 562.12, 562.13, 568.81, 569.3, 569.85, 569.86, 578.0, 578.1, 578.9  ICD-9-PCS: 44.43 | Gastrointestinal bleeding (GIB)  ICD-10-CM: I85.01, I85.11, K22.11, K22.6, K22.8, K25.0, K25.2, K25.4, K25.6, K26.0, K26.2, K26.4, K26.6, K27.0, K27.2, K27.4, K27.6, K28.0, K28.2, K28.4, K28.6, K29.01, K29.41, K29.51, K29.61, K29.21, K29.71, K29.91, K29.81, K31.811, K31.82, K55.21, K56.69, K57.01, K57.11, K57.13, K57.41, K57.51, K57.53, K57.81, K57.93, K57.21, K57.31, K57.91, K57.33, K66.1, K62.5, K55.21, K63.81, K92.0, K92.1, K92.2, I85.01, I8511  ICD-10-PCS: 0W3P8ZZ | Major bleeding, a composite of GIB, ICH or bleeding at key anatomical sites, was identified by ≥ 1 inpatient claim with diagnosis codes in the primary position for major bleeding or procedure to manage a bleeding event.  CRNMB - the secondary safety outcome – was identified using the same diagnosis codes as major bleeding, but present in the secondary position in inpatient claims or any position in outpatient claims | ≥ 89% | ^11–13^ |
|  | Intracranial hemorrhage (ICH)  ICD-9-CM: 430.x, 431.x, 432.x, 852.0x, 852.2x, 852.4x, 853.x | Intracranial hemorrhage (ICH)  ICD-10-CM codes for ICH: I60.x, I61.x, I62.0x, I62.1, I62.9, S06.6x, S06.5x, S06.4x, S06.36x |  |  |  |
|  | Bleeding at other critical anatomical sites  ICD-9-CM: 078.6, 246.3, 285.1, 286.5, 336.1, 388.69, 360.43, 362.43, 362.81, 363.61, 363.62, 363.72, 364.41, 372.72, 376.32, 377.42, 379.23, 423.0, 459.0, 599.7, 599.70, 599.71, 602.1, 621.4, 626.2, 626.5, 626.7, 626.8, 626.9, 640.8x, 641.8x, 641.9x, 641.3x, 641.1x, 666.1x, 719.1x, 782.7, 784.7, 784.8, 866.01, 866.11, 790.01, 958.2, 998.1, 998.11, 998.12  ICD-9-PCS: 99.04 | Bleeding at other critical sites  ICD-10-CM: A98.5, D62, D68.312, D68.318, D68.32, G95.19, H92.2x, H44.81x, H35.73x, H35.6x, H31.30x, H31.31x, H31.41x, H21.0x, H11.3x, H05.23x, H47.02x, H43.1x, I31.2, R58, R31.9, R31.0, N42.1, N85.7, N92.0, N92.3, N93.0, N93.8, N92.6, N93.9, O20.8x, O46.x, O72.x, M25.0x, R23.3, R04.0, R04.1, S37.019, S37.029, S37.019, S37.029, R71.0, T79.2x, D78.2x, E36.0x, E89.1x, G97.3x, G97.5x, H59.1x, H59.3x, H95.2x, H95.4x, I97.4x, I97.6x, J95.6x, J95.83x, K91.6x, K91.84x, L76.0x, L76.2x, M96.8x, N99.6x, N99.82x  ICD-10-PCS: 30230N1, 30230P1, 30233N1, 30233P1, 30240N1, 30240P1, 30243N1, 30243P1, 30250N1, 30250P1, 30253N1, 30253P1, 30260N1, 30260P1, 30263N1, 30263P1 |  |  |  |

Abbreviations. GIB, gastrointestinal bleeding; ICH; intracranial hemorrhage; PPV, positive predictive value; VTE, venous thromboembolism.

**Appendix Table 3: Adjusted covariates and time of assessment**

| **Covariates** | **Time of assessment** | **Additional information** |
| --- | --- | --- |
| **Demographic characteristics** | | |
| Age, gender, and geographic region | On index date | Age was analyzed as a continuous and categorical variable, while gender was categorized into either male or female. Geographic states were broadly categorized into 4 main regions: (a) Northeast; (b) Midwest; (c) South; and (d) West. We further added two categories: Puerto Rico and others/unknown to account for enrollees with missing or unknown geographic information. |
| Type of index VTE | Diagnosis for VTE occurred within 30 days prior of the index date | The type of index VTE event, whether deep vein thrombosis or pulmonary embolism, was assessed from the inpatient and outpatient data. The earliest encounter in medical records of eligible enrollees was considered the index VTE event. |
| **Comorbid conditions and lifestyle factors** | | |
| Falls, fractures involving the lower extremities, heart failure, other ischemic diseases, hypertension, diabetes, hyperlipidemia, obesity, acute or chronic renal disease, cancer, acute myocardial infarction, heart failure, other ischemic disease, peptic ulcer disease, smoking, provoked VTE | Within 12 months of index date, unless otherwise specified | Chronic conditions and lifestyle factors were assessed using information available in medical inpatient and outpatient data, and categorized as yes or no. Provoked VTE, assessed within three months of the index date was defined by the presence of any of the following transient or persistent risk factors for VTE: (a) hospitalization for ≥ 3 days, (b) receipt of estrogen therapy, or (c) medical encounters indicative of trauma, fracture, surgery or active cancer within three months of index VTE.^14^ Individuals that met any of these defining criteria were categorized as provoked cases, and otherwise as unprovoked VTE cases. |
| **Concomitant drug use** | | |
| ACEI, ARBs, statins, non-statin lipid lowering drugs, loop diuretics, potassium-sparing diuretics, thiazide diuretics, beta blockers, calcium channel blockers, nitrates, digoxin, metformin, sulfonylureas, insulin, other anti-diabetic drugs, aspirin, non-aspirin antiplatelet agents, NSAIDs, estrogen therapy, COX-2 inhibitors, H2 antagonists, proton pump inhibitors, cytochrome P450 3A enzymes inhibitors or inducers of (moderate or strong), P-glycoprotein inducers or inhibitors | Within 12 months of index date | Out-patient fill of relevant medication was assessed from prescription data of eligible enrollees using generic or brand names, or their corresponding National Drug Codes. P-glycoprotein and cytochrome P450 enzymes inducers or inhibitors were identified using the 2020 FDA Clinical Drug Interaction Studies guidance for industry^15^ and the 2018 European Heart Rhythm Association Practice Guide on the use of non-vitamin K antagonist oral anticoagulants in patients with atrial fibrillation^16^ |
| **Others** | | |
| HAS-BLED (Hypertension, Abnormal renal/liver function, Stroke, Bleeding history or predisposition, Labile international normalized ratio, Elderly (>65 years), Drugs/alcohol concomitantly) score | Within 12 months of index date | We modified the HAS-BLED risk score^17^ to account for the lack of labile international normalized ratio in our data. Individuals received one point each for the presence of hypertension, chronic liver disease, chronic kidney disease, stroke, prior history of bleeding, elderly age (> 65 years), concomitant use of aspirin or NSAIDs, and history of alcohol use disorders. Per eligibility criteria, all patients had a history of chronic liver disease. As such, the minimum modified HAS-BLED score in the current study was 1. The modified HAS-BLED score for included patients was analyzed as a continuous variable. |
| The claims-based frailty index reported by Kim et al | Within 12 months of index date | We followed the algorithm reported by Kim et al^18^ to quantitatively assess frailty status in included patients. The claims-based frailty index score was analyzed as a continuous variable. |

Abbreviations. ACEI, angiotensin-converting-enzyme inhibitors; ARBs, angiotensin-2 receptor blockers; COX-2, cyclooxygenase-2; FDA, Food and Drug Administration; HAS-BLED, Hypertension, Abnormal renal/liver function, Stroke, Bleeding history or predisposition, Labile international normalized ratio, Elderly (>65 years), Drugs/alcohol concomitantly score; NSAIDs, non-steroidal anti-inflammatory drugs; VTE, venous thromboembolism.

**Appendix Table 4: Distribution of treatment groups in patients with acute venous thromboembolism and chronic liver disease by treatment discontinuation and switching.**

| **Characteristics** | **Apixaban**  **(895, 10.6%)** | **Dabigatran**  **(83, 1.0%)** | **Rivaroxaban**  **(2161, 25.5%)** | **Edoxaban**  **(1, 0.01%)** | **Warfarin**  **(5337, 63%)** | **Total**  **(8477, 100.0%)** |
| --- | --- | --- | --- | --- | --- | --- |
| **Discontinued index drug** | 666 (11.2) | 60 (1.0) | 1555 (26.2) | 1 (0.02) | 3645 (61.5) | 5927 (100.0) |
| **Switched to comparator drug (n = 505)** |  |  |  |  |  |  |
| Apixaban | 0 | 5 (10.6) | 18 (38.3) | 0 | 24 (51.1) | 47 (9.3) |
| Dabigatran | 3 (27.3) | 0 | 3 (27.3) | 0 | 5 (45.4) | 11 (2.2) |
| Rivaroxaban | 31 (32.6) | 7 (7.4) | 0 | 0 | 57 (60.0) | 95 (18.8) |
| Edoxaban | 0 | 0 | 0 | 0 | 0 | 0 |
| Warfarin | 85 (24.2) | 22 (6.2) | 245 (69.6) | 0 | 0 | 352 (69.7) |

**Appendix Table 5. Baseline characteristics of patients with acute venous thromboembolism and chronic liver disease initiating DOACs versus warfarin before and after propensity score matching**

|  | **Before PSM, subjects n (%)** | | | **After PSM, subjects n (%)** | | |
| --- | --- | --- | --- | --- | --- | --- |
|  | **DOAC users**  **(n=3140, 37.0%)** | **Warfarin users**  **(n=5337, 63%)** | **Std diff.** | **DOAC users**  **(n = 2361, 50%)** | **Warfarin users**  **(n = 2361, 50%)** | **Std diff.** |
| **Characteristics*** |  |  |  |  |  |  |
| **Demographic characteristics** |  |  |  |  |  |  |
| Mean age (SD), years | 63 (13.5) | 63.6 (13.7) | -0.05 | 63.8 (13.3) | 64.1 (13.7) | -0.02 |
| Age category, ≥ 65 years | 1529 (48.7) | 2724 (51) | -0.05 | 1206 (51.1) | 1238 (52.4) | -0.03 |
| Female | 1607 (51.2) | 2777 (52) | -0.02 | 1217 (51.5) | 1224 (51.8) | -0.01 |
| **Index year** |  |  |  |  |  |  |
| 2011 | 6 (0.2) | 1035 (19.4) | -0.68 | 6 (0.3) | 6 (0.3) | 0.00 |
| 2012 | 19 (0.6) | 1120 (21) | -0.70 | 19 (0.8) | 19 (0.8) | 0.00 |
| 2013 | 222 (7.1) | 894 (16.8) | -0.30 | 222 (9.4) | 282 (11.9) | -0.08 |
| 2014 | 376 (12) | 712 (13.3) | -0.04 | 371 (15.7) | 518 (21.9) | -0.16 |
| 2015 | 496 (15.8) | 572 (10.7) | 0.15 | 450 (19.1) | 537 (22.7) | -0.09 |
| 2016 | 879 (28) | 570 (10.7) | 0.45 | 619 (26.2) | 565 (23.9) | 0.05 |
| 2017 | 1142 (36.4) | 434 (8.1) | 0.72 | 674 (28.5) | 434 (18.4) | 0.24 |
| **Geographic region** |  |  |  |  |  |  |
| Northeast | 351 (11.2) | 526 (9.9) | 0.04 | 264 (11.2) | 267 (11.3) | 0.00 |
| Midwest | 729 (23.2) | 1450 (27.2) | -0.09 | 563 (23.8) | 631 (26.7) | -0.07 |
| South | 1409 (44.9) | 2053 (38.5) | 0.13 | 986 (41.8) | 902 (38.2) | 0.07 |
| West | 643 (20.5) | 1275 (23.9) | -0.08 | 541 (22.9) | 554 (23.5) | -0.01 |
| Others | 8 (0.3) | 33 (0.6) | -0.06 | 7 (0.3) | 7 (0.3) | 0.00 |
| **Type of index VTE episode** |  |  |  |  |  |  |
| DVT | 1977 (63.0) | 3477 (65.1) | -0.05 | 1512 (64) | 1506 (63.8) | 0.01 |
| PE | 1163 (37.0) | 1860 (34.9) | 0.03 | 1086 (46) | 1091 (46.2) | 0.00 |
| **Comorbid conditions and lifestyle factors** |  |  |  |  |  |  |
| Hypertension | 2231 (71.1) | 3874 (72.6) | -0.03 | 1699 (72) | 1702 (72.1) | 0.00 |
| Diabetes | 1048 (33.4) | 1914 (35.9) | -0.05 | 778 (33) | 808 (34.2) | -0.03 |
| Hyperlipidemia | 1795 (57.2) | 3072 (57.6) | -0.01 | 1336 (56.6) | 1344 (56.9) | -0.01 |
| Obesity | 1086 (34.6) | 1509 (28.3) | 0.14 | 784 (33.2) | 770 (32.6) | 0.01 |
| Acute renal disease | 537 (17.1) | 1113 (20.9) | -0.10 | 443 (18.8) | 471 (19.9) | -0.03 |
| Chronic renal disease | 559 (17.8) | 1005 (18.8) | -0.03 | 434 (18.4) | 455 (19.3) | -0.02 |
| Acute myocardial infarction | 122 (3.9) | 259 (4.9) | -0.05 | 102 (4.3) | 120 (5.1) | -0.04 |
| Heart failure | 478 (15.2) | 1016 (19) | -0.10 | 384 (16.3) | 423 (17.9) | -0.04 |
| Other ischemic disease | 739 (23.5) | 1250 (23.4) | 0.00 | 562 (23.8) | 556 (23.5) | 0.01 |
| Peptic ulcer disease | 55 (1.8) | 103 (1.9) | -0.01 | 46 (1.9) | 51 (2.2) | -0.01 |
| Smoking | 1160 (36.9) | 1682 (31.5) | 0.11 | 851 (36) | 843 (35.7) | 0.01 |
| Falls | 266 (8.5) | 393 (7.4) | 0.04 | 204 (8.6) | 207 (8.8) | 0.00 |
| Fracture | 774 (24.6) | 907 (17) | 0.19 | 561 (23.8) | 510 (21.6) | 0.05 |
| Provoked VTE | 2228 (71) | 4243 (79.5) | -0.20 | 1721 (72.9) | 1782 (75.5) | -0.06 |
| Cancer | 1596 (50.8) | 2715 (50.9) | 0.00 | 1189 (50.4) | 1166 (49.4) | 0.02 |
| **Medication history** |  |  |  |  |  |  |
| ARBs | 609 (19.4) | 944 (17.7) | 0.04 | 449 (19) | 430 (18.2) | 0.02 |
| ACEI | 888 (28.3) | 1732 (32.5) | -0.09 | 695 (29.4) | 733 (31) | -0.04 |
| Statins | 1209 (38.5) | 2066 (38.7) | 0.00 | 907 (38.4) | 913 (38.7) | -0.01 |
| Non-statin lipid lowering drugs | 312 (9.9) | 597 (11.2) | -0.04 | 235 (10) | 229 (9.7) | 0.01 |
| Loop diuretics | 511 (16.3) | 1029 (19.3) | -0.08 | 412 (17.5) | 447 (18.9) | -0.04 |
| Potassium-sparing diuretics | 270 (8.6) | 514 (9.6) | -0.04 | 213 (9) | 207 (8.8) | 0.01 |
| Thiazide diuretics | 363 (11.6) | 707 (13.2) | -0.05 | 272 (11.5) | 272 (11.5) | 0.00 |
| Beta-blockers | 942 (30) | 1786 (33.5) | -0.07 | 729 (30.9) | 757 (32.1) | -0.03 |
| Calcium channel blockers | 745 (23.7) | 1330 (24.9) | -0.03 | 581 (24.6) | 581 (24.6) | 0.00 |
| Metformin | 481 (15.3) | 844 (15.8) | -0.01 | 348 (14.7) | 358 (15.2) | -0.01 |
| Sulfonylureas | 236 (7.5) | 577 (10.8) | -0.11 | 189 (8) | 209 (8.9) | -0.03 |
| Insulin | 274 (8.7) | 556 (10.4) | -0.06 | 226 (9.6) | 234 (9.9) | -0.01 |
| Other antidiabetic drugs | 189 (6) | 316 (5.9) | 0.00 | 130 (5.5) | 141 (6) | -0.02 |
| Aspirin | 26 (0.8) | 41 (0.8) | 0.01 | 22 (0.9) | 22 (0.9) | 0.00 |
| Non-aspirin antiplatelets | 186 (5.9) | 361 (6.8) | -0.03 | 148 (6.3) | 159 (6.7) | -0.02 |
| NSAIDs | 897 (28.6) | 1375 (25.8) | 0.06 | 634 (26.9) | 623 (26.4) | 0.01 |
| COX-2 inhibitors | 93 (3) | 165 (3.1) | -0.01 | 71 (3) | 67 (2.8) | 0.01 |
| Other anticoagulants | 214 (6.8) | 405 (7.6) | -0.03 | 183 (7.8) | 183 (7.8) | 0.00 |
| Histamine H-2 antagonists | 181 (5.8) | 311 (5.8) | 0.00 | 140 (5.9) | 148 (6.3) | -0.01 |
| Proton pump inhibitors | 1221 (38.9) | 2076 (38.9) | 0.00 | 908 (38.5) | 940 (39.8) | -0.03 |
| P-glycoprotein inhibitors | 992 (31.6) | 1798 (33.7) | -0.04 | 743 (31.5) | 770 (32.6) | -0.02 |
| P-glycoprotein inducers | 47 (1.5) | 96 (1.8) | -0.02 | 38 (1.6) | 43 (1.8) | -0.02 |
| Cytochrome P450 3A inhibitors | 125 (4) | 305 (5.7) | -0.08 | 92 (3.9) | 97 (4.1) | -0.01 |
| Cytochrome P450 3A inducers | 62 (2) | 116 (2.2) | -0.01 | 49 (2.1) | 52 (2.2) | -0.01 |
| Estrogen therapy | 192 (6.1) | 309 (5.8) | 0.01 | 139 (5.9) | 134 (5.7) | 0.01 |
| **Others** |  |  |  |  |  |  |
| Kim et al CFI,^18^ mean (SD) | 0.2 (0.1) | 0.2 (0.1) | -0.12 | 0.2 (0.1) | 0.2 (0.1) | -0.06 |
| HAS-BLED score, mean (SD) | 3.5 (1.3) | 3.6 (1.3) | -0.01 | 3.6 (1.3) | 3.6 (1.3) | -0.01 |
| **Follow-up time, days** |  |  |  |  |  |  |
| Mean (SD) | 88.4 (66.0) | 92.2 (66.6) | - | 89.2 (66.3) | 88.8 (67.1) | - |
| Median (Q1, Q3) | 74 (30, 163) | 81 (30, 175) | - | 74 (30, 163) | 75 (30, 170) | - |

^*^ Unless otherwise specified, baseline characteristics are presented as counts and percentages. Q1 and Q3 represents the lower and upper quartile respectively. Index year and follow-up time was not included in propensity score model as matching variables. Abbreviations. ACEI, angiotensin-converting-enzyme inhibitors; ARBs, angiotensin-2 receptor blockers; CFI, claims-based frailty index; COX-2, cyclooxygenase-2; DOACs, direct oral anticoagulants; DVT, deep vein thrombosis; HAS-BLED, Hypertension, Abnormal renal/liver function, Stroke, Bleeding history or predisposition, Labile international normalized ratio, Elderly (>65 years), Drugs/alcohol concomitantly score; NSAIDs, non-steroidal anti-inflammatory drugs; PSM, propensity score matching; PE, pulmonary embolism; standard deviation, SD; VTE, venous thromboembolism

**Appendix Table 6: Distribution of the types of chronic liver disease among patients with acute venous thromboembolism and chronic liver disease**

| **Chronic liver disease type** | **Before PSM, patients n (%)** | | |
| --- | --- | --- | --- |
|  | **DOACs**  **(3140, 37.0)** | **Warfarin**  **(5337, 63.0)** | **Total**  **(8477, 100.0)** |
| NAFLD/NASH | 1156 (36.8) | 1676 (31.4) | 2832 (33.4) |
| NAFLD/NASH only | 903 (28.8) | 1282 (24.0) | 2185 (25.8) |
| Cirrhosis | 810 (25.8) | 1639 (30.7) | 2449 (28.9) |
| Compensated | 182 (5.8) | 453 (8.5) | 635 (7.5) |
| Compensated only | 98 (3.1) | 238 (4.5) | 336 (4.0) |
| Decompensated | 468 (14.9) | 1053 (19.7) | 1521 (18.0) |
| Decompensated only | 469 (14.9) | 981 (18.4) | 1450 (17.1) |
| Viral hepatitis | 255 (8.1) | 430 (8.1) | 685 (8.1) |
| Liver cancer | 147 (4.7) | 273 (5.1) | 420 (5.0) |
| Liver failure | 94 (3.0) | 276 (5.2) | 370 (4.4) |
| Alcoholic liver disease | 92 (2.9) | 215 (4.0) | 307 (3.6) |
| Liver diseases of genetic causes |  |  |  |
| Hereditary hemochromatosis | 90 (2.9) | 135 (2.5) | 225 (2.7) |
| Alpha-1-antitrypsin deficiency | 9 (0.3) | 14 (0.3) | 23 (0.3) |
| Wilson | 5 (0.2) | 8 (0.2) | 13 (0.2) |
| Liver diseases of autoimmune causes |  |  |  |
| Autoimmune hepatitis | 64 (2.0) | 137 (2.6) | 201 (2.4) |
| Chronic hepatitis | 34 (1.1) | 105 (2.0) | 139 (1.6) |
| Budd-Chiari disease | 23 (0.7) | 59 (1.1) | 82 (1.0) |
| Liver transplantation | 13 (0.4) | 43 (0.8) | 56 (0.7) |
| Secondary or unspecified biliary cirrhosis | 12 (0.4) | 35 (0.7) | 47 (0.6) |
| Others ^*^ | 336 (10.7) | 292 (5.5) | 628 (7.4) |

^*^ Others include hepatitis fibrosis or sclerosis or fibrosis with sclerosis; portal vein thrombosis; inflammatory liver disease, unspecified; liver disease, unspecified Abbreviations. NAFLD; non-alcoholic fatty liver disease, NASH; non-alcoholic steatohepatitis.

**Appendix Table 7: Baseline characteristics of patients with acute venous thromboembolism and chronic liver disease initiating apixaban versus warfarin before and after propensity score matching.**

| **Characteristics*** | **Before PSM, subjects n (%)** | | | **After PSM, subjects n (%)** | | |
| --- | --- | --- | --- | --- | --- | --- |
|  | **Apixaban users**  **(n=895, 14.4%)** | **Warfarin users**  **(n=5337, 85.6%)** | **Std diff.** | **Apixaban users**  **(n = 895, 50%)** | **Warfarin users**  **(n = 895, 50%)** | **Std diff.** |
| **Demographic characteristics** |  |  |  |  |  |  |
| Mean age (SD), years | 66 (13.3) | 63.6 (13.7) | 0.18 | 66 (13.3) | 66.4 (13.1) | -0.03 |
| Age category, ≥ 65 years | 520 (58.1) | 2724 (51) | 0.14 | 520 (58.1) | 527 (58.9) | -0.02 |
| Female | 475 (53.1) | 2777 (52) | 0.02 | 475 (53.1) | 480 (53.6) | -0.01 |
| **Index year** |  |  |  |  |  |  |
| 2011 | 0 (0) | 1035 (19.4) | - | 0 (0) | 0 (0) | - |
| 2012 | 0 (0) | 1120 (21) | - | 0 (0) | 0 (0) | - |
| 2013 | 0 (0) | 894 (16.8) | - | 0 (0) | 0 (0) | - |
| 2014 | 14 (1.6) | 712 (13.3) | - | 14 (1.6) | 14 (1.6) | 0.00 |
| 2015 | 97 (10.8) | 572 (10.7) | - | 97 (10.8) | 96 (10.7) | 0.00 |
| 2016 | 302 (33.7) | 570 (10.7) | - | 302 (33.7) | 374 (41.8) | -0.17 |
| 2017 | 482 (53.9) | 434 (8.1) | - | 482 (53.9) | 411 (45.9) | 0.16 |
| **Geographic region** |  |  |  |  |  |  |
| Northeast | 87 (9.7) | 526 (9.9) | 0.00 | 87 (9.7) | 97 (10.8) | -0.04 |
| Midwest | 187 (20.9) | 1450 (27.2) | -0.15 | 187 (20.9) | 233 (26) | -0.11 |
| South | 433 (48.4) | 2053 (38.5) | 0.20 | 433 (48.4) | 346 (38.7) | 0.20 |
| West | 186 (20.8) | 1275 (23.9) | -0.07 | 186 (20.8) | 217 (24.2) | -0.08 |
| Others | 2 (0.2) | 33 (0.6) | -0.06 | 2 (0.2) | 2 (0.2) | 0.00 |
| **Type of index VTE episode** |  |  |  |  |  |  |
| DVT | 524 (58.6) | 3477 (65.1) | -0.14 | 524 (58.5) | 535 (59.8) | -0.03 |
| PE | 371 (41.4) | 1860 (34.9) | 0.13 | 463 (51.7) | 446 (49.8) | 0.04 |
| **Comorbid conditions and lifestyle factors** |  |  |  |  |  |  |
| Hypertension | 680 (76) | 3874 (72.6) | 0.08 | 680 (76) | 672 (75.1) | 0.02 |
| Diabetes | 319 (35.6) | 1914 (35.9) | 0.00 | 319 (35.6) | 330 (36.9) | -0.03 |
| Hyperlipidemia | 548 (61.2) | 3072 (57.6) | 0.07 | 548 (61.2) | 518 (57.9) | 0.07 |
| Obesity | 320 (35.8) | 1509 (28.3) | 0.16 | 320 (35.8) | 319 (35.6) | 0.00 |
| Acute renal disease | 196 (21.9) | 1113 (20.9) | 0.03 | 196 (21.9) | 213 (23.8) | -0.05 |
| Chronic renal disease | 206 (23) | 1005 (18.8) | 0.10 | 206 (23) | 203 (22.7) | 0.01 |
| Acute myocardial infarction | 47 (5.3) | 259 (4.9) | 0.02 | 47 (5.3) | 57 (6.4) | -0.05 |
| Heart failure | 179 (20) | 1016 (19) | 0.02 | 179 (20) | 200 (22.3) | -0.06 |
| Other ischemic disease | 249 (27.8) | 1250 (23.4) | 0.10 | 249 (27.8) | 241 (26.9) | 0.02 |
| Peptic ulcer disease | 22 (2.5) | 103 (1.9) | 0.04 | 22 (2.5) | 27 (3) | -0.03 |
| Smoking | 350 (39.1) | 1682 (31.5) | 0.16 | 350 (39.1) | 360 (40.2) | -0.02 |
| Falls | 93 (10.4) | 393 (7.4) | 0.11 | 93 (10.4) | 105 (11.7) | -0.04 |
| Fracture | 278 (31.1) | 907 (17) | 0.33 | 278 (31.1) | 279 (31.2) | 0.00 |
| Provoked VTE | 659 (73.6) | 4243 (79.5) | -0.14 | 659 (73.6) | 680 (76) | -0.05 |
| Cancer | 447 (49.9) | 2715 (50.9) | -0.02 | 447 (49.9) | 445 (49.7) | 0.00 |
| **Medication history** |  |  |  |  |  |  |
| ARBs | 186 (20.8) | 944 (17.7) | 0.08 | 186 (20.8) | 176 (19.7) | 0.03 |
| ACEI | 261 (29.2) | 1732 (32.5) | -0.07 | 261 (29.2) | 268 (29.9) | -0.02 |
| Statins | 377 (42.1) | 2066 (38.7) | 0.07 | 377 (42.1) | 366 (40.9) | 0.02 |
| Non-statin lipid lowering drugs | 94 (10.5) | 597 (11.2) | -0.02 | 94 (10.5) | 81 (9.1) | 0.05 |
| Loop diuretics | 172 (19.2) | 1029 (19.3) | 0.00 | 172 (19.2) | 189 (21.1) | -0.05 |
| Potassium-sparing diuretics | 65 (7.3) | 514 (9.6) | -0.09 | 65 (7.3) | 71 (7.9) | -0.03 |
| Thiazide diuretics | 122 (13.6) | 707 (13.2) | 0.01 | 122 (13.6) | 107 (12) | 0.05 |
| Beta-blockers | 286 (32) | 1786 (33.5) | -0.03 | 286 (32) | 293 (32.7) | -0.02 |
| Calcium channel blockers | 238 (26.6) | 1330 (24.9) | 0.04 | 238 (26.6) | 229 (25.6) | 0.02 |
| Metformin | 137 (15.3) | 844 (15.8) | -0.01 | 137 (15.3) | 145 (16.2) | -0.02 |
| Sulfonylureas | 65 (7.3) | 577 (10.8) | -0.12 | 65 (7.3) | 71 (7.9) | -0.03 |
| Insulin | 88 (9.8) | 556 (10.4) | -0.02 | 88 (9.8) | 95 (10.6) | -0.03 |
| Other antidiabetic drugs | 55 (6.1) | 316 (5.9) | 0.01 | 55 (6.1) | 52 (5.8) | 0.01 |
| Aspirin | 5 (0.6) | 41 (0.8) | -0.03 | 5 (0.6) | 7 (0.8) | -0.03 |
| Non-aspirin antiplatelets | 59 (6.6) | 361 (6.8) | -0.01 | 59 (6.6) | 67 (7.5) | -0.03 |
| NSAIDs | 265 (29.6) | 1375 (25.8) | 0.09 | 265 (29.6) | 238 (26.6) | 0.07 |
| COX-2 inhibitors | 23 (2.6) | 165 (3.1) | -0.03 | 23 (2.6) | 22 (2.5) | 0.01 |
| Histamine H-2 antagonists | 70 (7.8) | 311 (5.8) | 0.08 | 48 (5.4) | 56 (6.3) | -0.04 |
| Proton pump inhibitors | 345 (38.5) | 2076 (38.9) | -0.01 | 70 (7.8) | 65 (7.3) | 0.02 |
| P-glycoprotein inhibitors | 285 (31.8) | 1798 (33.7) | -0.04 | 345 (38.5) | 339 (37.9) | 0.01 |
| P-glycoprotein inducers | 14 (1.6) | 96 (1.8) | -0.02 | 285 (31.8) | 270 (30.2) | 0.04 |
| Cytochrome P450 3A inhibitors | 36 (4) | 305 (5.7) | -0.08 | 14 (1.6) | 15 (1.7) | -0.01 |
| Cytochrome P450 3A inducers | 19 (2.1) | 116 (2.2) | 0.00 | 36 (4) | 33 (3.7) | 0.02 |
| Estrogen therapy | 48 (5.4) | 309 (5.8) | -0.02 | 19 (2.1) | 19 (2.1) | 0.00 |
| **Others** |  |  |  |  |  |  |
| Kim et al CFI,^18^ mean (SD) | 0.2 (0.1) | 0.2 (0.1) | 0.09 | 0.2 (0.1) | 0.2 (0.1) | -0.05 |
| HAS-BLED score, mean (SD) | 3.8 (1.3) | 3.6 (1.3) | 0.21 | 3.8 (1.3) | 3.8 (1.2) | 0.02 |

^*^ Unless otherwise specified, baseline characteristics are presented as counts and percentages. Q1 and Q3 represents the lower and upper quartile respectively. Index year was not included in propensity score model. Abbreviations. ACEI, angiotensin-converting-enzyme inhibitors; ARBs, angiotensin-2 receptor blockers; CFI, claims-based frailty index; COX-2, cyclooxygenase-2; DOACs, direct oral anticoagulants; DVT, deep vein thrombosis; HAS-BLED, Hypertension, Abnormal renal/liver function, Stroke, Bleeding history or predisposition, Labile international normalized ratio, Elderly (>65 years), Drugs/alcohol concomitantly score; NSAIDs, non-steroidal anti-inflammatory drugs; PSM, propensity score matching; PE, pulmonary embolism; standard deviation, SD; VTE, venous thromboembolism

**Appendix Table 8: Baseline characteristics of patients with acute venous thromboembolism and chronic liver disease initiating rivaroxaban versus warfarin before and after propensity score matching.**

| **Characteristics*** | **Before PSM, subjects n (%)** | | | **After PSM, subjects n (%)** | | |
| --- | --- | --- | --- | --- | --- | --- |
|  | **Rivaroxaban users**  **(n=2161, 28.8%)** | **Warfarin users**  **(n=5337, 71.2%)** | **Std diff.** | **Rivaroxaban users**  **(n = 2161, 50%)** | **Warfarin users**  **(n = 2161, 50%)** | **Std diff.** |
| **Demographic characteristics** |  |  |  |  |  |  |
| Mean age (SD), years | 61.7 (13.4) | 63.6 (13.7) | -0.14 | 61.7 (13.4) | 63.3 (13.9) | -0.09 |
| Age category, ≥ 65 years | 970 (44.9) | 2724 (51) | -0.12 | 970 (44.9) | 1088 (50.3) | -0.11 |
| Female | 1093 (50.6) | 2777 (52) | -0.03 | 1093 (50.6) | 1113 (51.5) | -0.02 |
| **Index year** |  |  |  |  |  |  |
| 2011 | 0 (0) | 1035 (19.4) | - | 0 (0) | 0 (0) | - |
| 2012 | 17 (0.8) | 1120 (21) | - | 17 (0.8) | 16 (0.7) | 0.01 |
| 2013 | 219 (10.1) | 894 (16.8) | - | 219 (10.1) | 267 (12.4) | -0.07 |
| 2014 | 358 (16.6) | 712 (13.3) | - | 358 (16.6) | 477 (22.1) | -0.11 |
| 2015 | 390 (18) | 572 (10.7) | - | 390 (18) | 465 (21.5) | -0.09 |
| 2016 | 541 (25) | 570 (10.7) | - | 541 (25) | 519 (24) | 0.02 |
| 2017 | 636 (29.4) | 434 (8.1) | - | 636 (29.4) | 417 (19.3) | 0.24 |
| **Geographic region** |  |  |  |  |  |  |
| Northeast | 253 (11.7) | 526 (9.9) | 0.06 | 253 (11.7) | 246 (11.4) | 0.01 |
| Midwest | 524 (24.2) | 1450 (27.2) | -0.07 | 524 (24.2) | 582 (26.9) | -0.06 |
| South | 940 (43.5) | 2053 (38.5) | 0.10 | 940 (43.5) | 837 (38.7) | 0.10 |
| West | 438 (20.3) | 1275 (23.9) | -0.09 | 438 (20.3) | 489 (22.6) | -0.06 |
| Others | 6 (0.3) | 33 (0.6) | -0.05 | 6 (0.3) | 7 (0.3) | -0.01 |
| **Type of index VTE episode** |  |  |  |  |  |  |
| DVT | 1399 (64.7) | 3477 (65.1) | -0.01 | 1399 (64.7) | 1399 (64.7) | 0.00 |
| PE | 762 (35.3) | 1860 (34.9) | -0.01 | 970 (44.9) | 977 (45.2) | -0.01 |
| **Comorbid conditions and lifestyle factors** |  |  |  |  |  |  |
| Hypertension | 1486 (68.8) | 3874 (72.6) | -0.08 | 1486 (68.8) | 1542 (71.4) | -0.06 |
| Diabetes | 700 (32.4) | 1914 (35.9) | -0.07 | 700 (32.4) | 722 (33.4) | -0.02 |
| Hyperlipidemia | 1196 (55.3) | 3072 (57.6) | -0.04 | 1196 (55.3) | 1214 (56.2) | -0.02 |
| Obesity | 733 (33.9) | 1509 (28.3) | 0.12 | 733 (33.9) | 703 (32.5) | 0.03 |
| Acute renal disease | 325 (15) | 1113 (20.9) | -0.15 | 325 (15) | 388 (18) | -0.08 |
| Chronic renal disease | 336 (15.5) | 1005 (18.8) | -0.09 | 336 (15.5) | 383 (17.7) | -0.06 |
| Acute myocardial infarction | 75 (3.5) | 259 (4.9) | -0.07 | 75 (3.5) | 97 (4.5) | -0.05 |
| Heart failure | 286 (13.2) | 1016 (19) | -0.16 | 286 (13.2) | 331 (15.3) | -0.06 |
| Other ischemic disease | 469 (21.7) | 1250 (23.4) | -0.04 | 469 (21.7) | 482 (22.3) | -0.01 |
| Peptic ulcer disease | 31 (1.4) | 103 (1.9) | -0.04 | 31 (1.4) | 40 (1.9) | -0.03 |
| Smoking | 775 (35.9) | 1682 (31.5) | 0.09 | 775 (35.9) | 750 (34.7) | 0.02 |
| Falls | 163 (7.5) | 393 (7.4) | 0.01 | 163 (7.5) | 176 (8.1) | -0.02 |
| Fracture | 467 (21.6) | 907 (17) | 0.12 | 467 (21.6) | 447 (20.7) | 0.02 |
| Provoked VTE | 1502 (69.5) | 4243 (79.5) | -0.23 | 1502 (69.5) | 1611 (74.5) | -0.11 |
| Cancer | 1105 (51.1) | 2715 (50.9) | 0.01 | 1105 (51.1) | 1086 (50.3) | 0.02 |
| **Medication history** |  |  |  |  |  |  |
| ARBs | 403 (18.6) | 944 (17.7) | 0.02 | 403 (18.6) | 402 (18.6) | 0.00 |
| ACEI | 599 (27.7) | 1732 (32.5) | -0.10 | 599 (27.7) | 639 (29.6) | -0.04 |
| Statins | 795 (36.8) | 2066 (38.7) | -0.04 | 795 (36.8) | 817 (37.8) | -0.02 |
| Non-statin lipid lowering drugs | 209 (9.7) | 597 (11.2) | -0.05 | 209 (9.7) | 189 (8.7) | 0.03 |
| Loop diuretics | 317 (14.7) | 1029 (19.3) | -0.12 | 317 (14.7) | 359 (16.6) | -0.05 |
| Potassium-sparing diuretics | 196 (9.1) | 514 (9.6) | -0.02 | 196 (9.1) | 198 (9.2) | 0.00 |
| Thiazide diuretics | 236 (10.9) | 707 (13.2) | -0.07 | 236 (10.9) | 236 (10.9) | 0.00 |
| Beta-blockers | 621 (28.7) | 1786 (33.5) | -0.10 | 621 (28.7) | 643 (29.8) | -0.02 |
| Calcium channel blockers | 484 (22.4) | 1330 (24.9) | -0.06 | 484 (22.4) | 516 (23.9) | -0.04 |
| Metformin | 329 (15.2) | 844 (15.8) | -0.02 | 329 (15.2) | 322 (14.9) | 0.01 |
| Sulfonylureas | 164 (7.6) | 577 (10.8) | -0.11 | 164 (7.6) | 175 (8.1) | -0.02 |
| Insulin | 179 (8.3) | 556 (10.4) | -0.07 | 179 (8.3) | 202 (9.3) | -0.04 |
| Other antidiabetic drugs | 130 (6) | 316 (5.9) | 0.00 | 130 (6) | 120 (5.6) | 0.02 |
| Aspirin | 20 (0.9) | 41 (0.8) | 0.02 | 20 (0.9) | 23 (1.1) | -0.01 |
| Non-aspirin antiplatelets | 125 (5.8) | 361 (6.8) | -0.04 | 125 (5.8) | 137 (6.3) | -0.02 |
| NSAIDs | 606 (28) | 1375 (25.8) | 0.05 | 606 (28) | 579 (26.8) | 0.03 |
| COX-2 inhibitors | 67 (3.1) | 165 (3.1) | 0.00 | 67 (3.1) | 58 (2.7) | 0.02 |
| Histamine H-2 antagonists | 108 (5) | 311 (5.8) | -0.04 | 161 (7.5) | 154 (7.1) | 0.01 |
| Proton pump inhibitors | 846 (39.1) | 2076 (38.9) | 0.01 | 108 (5) | 124 (5.7) | -0.03 |
| P-glycoprotein inhibitors | 678 (31.4) | 1798 (33.7) | -0.05 | 846 (39.1) | 847 (39.2) | 0.00 |
| P-glycoprotein inducers | 32 (1.5) | 96 (1.8) | -0.03 | 678 (31.4) | 704 (32.6) | -0.03 |
| Cytochrome P450 3A inhibitors | 82 (3.8) | 305 (5.7) | -0.09 | 32 (1.5) | 36 (1.7) | -0.01 |
| Cytochrome P450 3A inducers | 42 (1.9) | 116 (2.2) | -0.02 | 82 (3.8) | 91 (4.2) | -0.02 |
| Estrogen therapy | 141 (6.5) | 309 (5.8) | 0.03 | 42 (1.9) | 44 (2) | -0.01 |
| **Others** |  |  |  |  |  |  |
| Kim et al CFI,^18^ mean (SD) | 0.2 (0.1) | 0.2 (0.1) | -0.21 | 0.2 (0.1) | 0.2 (0.1) | -0.05 |
| HAS-BLED score, mean (SD) | 3.4 (1.3) | 3.6 (1.3) | -0.10 | 3.4 (1.3) | 3.5 (1.3) | -0.08 |

^*^ Unless otherwise specified, baseline characteristics are presented as counts and percentages. Q1 and Q3 represents the lower and upper quartile respectively. Index year was not included in propensity score model. Abbreviations. ACEI, angiotensin-converting-enzyme inhibitors; ARBs, angiotensin-2 receptor blockers; CFI, claims-based frailty index; COX-2, cyclooxygenase-2; DOACs, direct oral anticoagulants; DVT, deep vein thrombosis; HAS-BLED, Hypertension, Abnormal renal/liver function, Stroke, Bleeding history or predisposition, Labile international normalized ratio, Elderly (>65 years), Drugs/alcohol concomitantly score; NSAIDs, non-steroidal anti-inflammatory drugs; PSM, propensity score matching; PE, pulmonary embolism; standard deviation, SD; VTE, venous thromboembolism

**Appendix Table 9: Baseline characteristics of patients with acute venous thromboembolism and chronic liver disease initiating apixaban versus rivaroxaban before and after propensity score matching.**

| **Characteristics*** | **Before PSM, subjects n (%)** | | | **After PSM, subjects n (%)** | | |
| --- | --- | --- | --- | --- | --- | --- |
|  | **Apixaban users**  **(n=895, 29.3%)** | **Rivaroxaban users**  **(n=2161, 70.7%)** | **Std diff.** | **Apixaban users**  **(n = 895, 50%)** | **Rivaroxaban users**  **(n = 895, 50%)** | **Std diff.** |
| **Demographic characteristics** |  |  |  |  |  |  |
| Mean age (SD), years | 66 (13.3) | 61.7 (13.4) | 0.32 | 66 (13.3) | 65.1 (12.4) | 0.07 |
| Age category, ≥ 65 years | 520 (58.1) | 970 (44.9) | 0.27 | 520 (58.1) | 494 (55.2) | 0.06 |
| Female | 475 (53.1) | 1093 (50.6) | 0.05 | 475 (53.1) | 457 (51.1) | 0.04 |
| **Index year** |  |  |  |  |  |  |
| 2012 | 0 (0) | 17 (0.8) | - | 0 (0) | 0 (0) |  |
| 2013 | 0 (0) | 219 (10.1) | - | 0 (0) | 0 (0) |  |
| 2014 | 14 (1.6) | 358 (16.6) | - | 14 (1.6) | 14 (1.6) | 0.00 |
| 2015 | 97 (10.8) | 390 (18) | - | 97 (10.8) | 99 (11.1) | -0.01 |
| 2016 | 302 (33.7) | 541 (25) | - | 302 (33.7) | 312 (34.9) | -0.02 |
| 2017 | 482 (53.9) | 636 (29.4) | - | 482 (53.9) | 470 (52.5) | 0.03 |
| **Geographic region** |  |  |  |  |  |  |
| Northeast | 87 (9.7) | 253 (11.7) | -0.06 | 87 (9.7) | 88 (9.8) | 0.00 |
| Midwest | 187 (20.9) | 524 (24.2) | -0.08 | 187 (20.9) | 203 (22.7) | -0.04 |
| South | 433 (48.4) | 940 (43.5) | 0.10 | 433 (48.4) | 416 (46.5) | 0.04 |
| West | 186 (20.8) | 438 (20.3) | 0.01 | 186 (20.8) | 186 (20.8) | 0.00 |
| Others | 2 (0.2) | 6 (0.3) | -0.01 | 2 (0.2) | 2 (0.2) | 0.00 |
| **Type of index VTE episode** |  |  |  |  |  |  |
| DVT | 524 (58.5) | 1399 (64.7) | -0.13 | 524 (58.5) | 522 (58.3) | 0.00 |
| PE | 371 (41.5) | 762 (35.3) | 0.14 | 463 (51.7) | 461 (51.5) | 0.00 |
| **Comorbid conditions and lifestyle factors** |  |  |  |  |  |  |
| Hypertension | 680 (76) | 1486 (68.8) | 0.16 | 680 (76) | 670 (74.9) | 0.03 |
| Diabetes | 319 (35.6) | 700 (32.4) | 0.07 | 319 (35.6) | 314 (35.1) | 0.01 |
| Hyperlipidemia | 548 (61.2) | 1196 (55.3) | 0.12 | 548 (61.2) | 540 (60.3) | 0.02 |
| Obesity | 320 (35.8) | 733 (33.9) | 0.04 | 320 (35.8) | 308 (34.4) | 0.03 |
| Acute renal disease | 196 (21.9) | 325 (15) | 0.18 | 196 (21.9) | 174 (19.4) | 0.06 |
| Chronic renal disease | 206 (23) | 336 (15.5) | 0.19 | 206 (23) | 193 (21.6) | 0.03 |
| Acute myocardial infarction | 47 (5.3) | 75 (3.5) | 0.09 | 47 (5.3) | 40 (4.5) | 0.04 |
| Heart failure | 179 (20) | 286 (13.2) | 0.18 | 179 (20) | 154 (17.2) | 0.07 |
| Other ischemic disease | 249 (27.8) | 469 (21.7) | 0.14 | 249 (27.8) | 226 (25.3) | 0.06 |
| Peptic ulcer disease | 22 (2.5) | 31 (1.4) | 0.07 | 22 (2.5) | 19 (2.1) | 0.02 |
| Smoking | 350 (39.1) | 775 (35.9) | 0.07 | 350 (39.1) | 363 (40.6) | -0.03 |
| Falls | 93 (10.4) | 163 (7.5) | 0.10 | 93 (10.4) | 95 (10.6) | -0.01 |
| Fracture | 278 (31.1) | 467 (21.6) | 0.22 | 278 (31.1) | 253 (28.3) | 0.06 |
| Provoked VTE | 659 (73.6) | 1502 (69.5) | 0.09 | 659 (73.6) | 655 (73.2) | 0.01 |
| Cancer | 447 (49.9) | 1105 (51.1) | -0.02 | 447 (49.9) | 446 (49.8) | 0.00 |
| **Medication history** |  |  |  |  |  |  |
| ARBs | 186 (20.8) | 403 (18.6) | 0.05 | 186 (20.8) | 176 (19.7) | 0.03 |
| ACEI | 261 (29.2) | 599 (27.7) | 0.03 | 261 (29.2) | 247 (27.6) | 0.03 |
| Statins | 377 (42.1) | 795 (36.8) | 0.11 | 377 (42.1) | 361 (40.3) | 0.04 |
| Non-statin lipid lowering drugs | 94 (10.5) | 209 (9.7) | 0.03 | 94 (10.5) | 92 (10.3) | 0.01 |
| Loop diuretics | 172 (19.2) | 317 (14.7) | 0.12 | 172 (19.2) | 156 (17.4) | 0.05 |
| Potassium-sparing diuretics | 65 (7.3) | 196 (9.1) | -0.07 | 65 (7.3) | 69 (7.7) | -0.02 |
| Thiazide diuretics | 122 (13.6) | 236 (10.9) | 0.08 | 122 (13.6) | 111 (12.4) | 0.04 |
| Beta-blockers | 286 (32) | 621 (28.7) | 0.07 | 286 (32) | 276 (30.8) | 0.02 |
| Calcium channel blockers | 238 (26.6) | 484 (22.4) | 0.10 | 238 (26.6) | 217 (24.2) | 0.05 |
| Metformin | 137 (15.3) | 329 (15.2) | 0.00 | 137 (15.3) | 139 (15.5) | -0.01 |
| Sulfonylureas | 65 (7.3) | 164 (7.6) | -0.01 | 65 (7.3) | 68 (7.6) | -0.01 |
| Insulin | 88 (9.8) | 179 (8.3) | 0.05 | 88 (9.8) | 84 (9.4) | 0.02 |
| Other antidiabetic drugs | 55 (6.1) | 130 (6) | 0.01 | 55 (6.1) | 55 (6.1) | 0.00 |
| Aspirin | 5 (0.6) | 20 (0.9) | -0.04 | 5 (0.6) | 7 (0.8) | -0.03 |
| Non-aspirin antiplatelets | 59 (6.6) | 125 (5.8) | 0.03 | 59 (6.6) | 55 (6.1) | 0.02 |
| NSAIDs | 265 (29.6) | 606 (28) | 0.03 | 265 (29.6) | 264 (29.5) | 0.00 |
| COX-2 inhibitors | 23 (2.6) | 67 (3.1) | -0.03 | 23 (2.6) | 26 (2.9) | -0.02 |
| Histamine H-2 antagonists | 70 (7.8) | 108 (5) | 0.12 | 70 (7.8) | 64 (7.2) | 0.03 |
| Proton pump inhibitors | 345 (38.5) | 846 (39.1) | -0.01 | 345 (38.5) | 339 (37.9) | 0.01 |
| P-glycoprotein inhibitors | 285 (31.8) | 678 (31.4) | 0.01 | 285 (31.8) | 285 (31.8) | 0.00 |
| P-glycoprotein inducers | 14 (1.6) | 32 (1.5) | 0.01 | 14 (1.6) | 15 (1.7) | -0.01 |
| Cytochrome P450 3A inhibitors | 36 (4) | 82 (3.8) | 0.01 | 36 (4) | 36 (4) | 0.00 |
| Cytochrome P450 3A inducers | 19 (2.1) | 42 (1.9) | 0.01 | 19 (2.1) | 20 (2.2) | -0.01 |
| Estrogen therapy | 48 (5.4) | 141 (6.5) | -0.05 | 48 (5.4) | 49 (5.5) | 0.00 |
| **Others** |  |  |  |  |  |  |
| Kim et al CFI,^18^ mean (SD) | 0.2 (0.1) | 0.2 (0.1) | 0.31 | 0.2 (0.1) | 0.2 (0.1) | 0.10 |
| HAS-BLED score, mean (SD) | 3.8 (1.3) | 3.4 (1.3) | 0.31 | 3.8 (1.3) | 3.7 (1.2) | 0.07 |

^*^ Unless otherwise specified, baseline characteristics are presented as counts and percentages. Q1 and Q3 represents the lower and upper quartile respectively. Index year was not included in propensity score model. Abbreviations. ACEI, angiotensin-converting-enzyme inhibitors; ARBs, angiotensin-2 receptor blockers; CFI, claims-based frailty index; COX-2, cyclooxygenase-2; DOACs, direct oral anticoagulants; DVT, deep vein thrombosis; HAS-BLED, Hypertension, Abnormal renal/liver function, Stroke, Bleeding history or predisposition, Labile international normalized ratio, Elderly (>65 years), Drugs/alcohol concomitantly score; NSAIDs, non-steroidal anti-inflammatory drugs; PSM, propensity score matching; PE, pulmonary embolism; standard deviation, SD; VTE, venous thromboembolism

**Appendix Table 10: Incidence rates and effect estimates for clinical outcomes by treatment groups using an alternative three month follow-up period.**

| **Clinical outcomes** | **Before PSM** | | | | **After PSM** | | | | **Unmatched HR**  **(95% CI)** | **PSM HR**  **(95% CI)** |
| --- | --- | --- | --- | --- | --- | --- | --- | --- | --- | --- |
|  | **Comparator** | | **Reference** | | **Comparator** | | **Reference** | |  |  |
|  | **n** | **IR/100**  **PY** | **n** | **IR/100**  **PY** | **n** | **IR/100**  **PY** | **n** | **IR/100**  **PY** |  |  |
| **Primary outcome** |  |  |  |  |  |  |  |  |  |  |
| Any DOAC vs warfarin | 139 | 24.8 | 370 | 37.2 | 95 | 21.4 | 171 | 40.8 | 0.66 (0.54, 0.80) | 0.55 (0.45, 0.68) |
| Apixaban vs warfarin | 32 | 20.7 | 370 | 37.4 | 32 | 22.1 | 65 | 45.2 | 0.54 (0.38, 0.78) | 0.46 (0.33, 0.66) |
| Rivaroxaban vs warfarin | 103 | 26.4 | 370 | 37.4 | 102 | 25.4 | 145 | 37.8 | 0.70 (0.56, 0.87) | 0.71 (0.58, 0.86) |
| Apixaban vs rivaroxaban | 32 | 20.8 | 103 | 26.4 | 32 | 20.6 | 44 | 29.0 | 0.77 (0.52, 1.15) | 0.74 (0.45, 1.23) |
| **Effectiveness outcomes** |  |  |  |  |  |  |  |  |  |  |
| **Recurrent VTE** |  |  |  |  |  |  |  |  |  |  |
| Any DOAC vs warfarin | 41 | 7.2 | 101 | 10.0 | 24 | 6.4 | 53 | 13.2 | 0.71 (0.50, 1.03) | 0.49 (0.33, 0.72) |
| Apixaban vs warfarin | 9 | 5.8 | 101 | 10.0 | 9 | 5.8 | 23 | 17.4 | 0.56 (0.28, 1.11) | 0.47 (0.25, 0.89) |
| Rivaroxaban vs warfarin | 29 | 7.4 | 101 | 10.0 | 29 | 8.4 | 43 | 12.0 | 0.73 (0.48, 1.10) | 0.80 (0.56, 1.14) |
| Apixaban vs rivaroxaban | 9 | 5.8 | 29 | 7.4 | 9 | 6.3 | 14 | 9.5 | 0.78 (0.37, 1.63) | 0.88 (0.35, 2.24) |
| **All-cause mortality** |  |  |  |  |  |  |  |  |  |  |
| Any DOAC vs warfarin | 161 | 28.5 | 248 | 24.4 | 114 | 28.1 | 117 | 27.7 | 1.16 (0.95, 1.41) | 0.97 (0.80, 1.18) |
| Apixaban vs warfarin | 65 | 42.2 | 248 | 24.4 | 65 | 43.5 | 61 | 38.1 | 1.69 (1.29, 2.22) | 1.19 (0.90, 1.56) |
| Rivaroxaban vs warfarin | 94 | 23.8 | 248 | 24.4 | 94 | 23.6 | 93 | 23.9 | 0.97 (0.77, 1.23) | 1.07 (0.85, 1.34) |
| Apixaban vs rivaroxaban | 65 | 42.2 | 94 | 23.8 | 65 | 47.6 | 58 | 41.9 | 1.70 (1.23, 2.34) | 1.20 (0.80, 1.81) |
| **Safety outcomes** |  |  |  |  |  |  |  |  |  |  |
| **Major bleeding** |  |  |  |  |  |  |  |  |  |  |
| Any DOAC vs warfarin | 101 | 17.9 | 282 | 28.3 | 72 | 15.7 | 126 | 29.2 | 0.63 (0.50, 0.79) | 0.55 (0.44, 0.70) |
| Apixaban vs warfarin | 23 | 14.9 | 282 | 28.3 | 23 | 14.7 | 45 | 29.0 | 0.51 (0.36, 0.78) | 0.43 (0.28, 0.65) |
| Rivaroxaban vs warfarin | 77 | 19.6 | 282 | 28.3 | 77 | 17.9 | 108 | 26.7 | 0.69 (0.53, 0.89) | 0.68 (0.54, 0.86) |
| Apixaban vs rivaroxaban | 23 | 14.9 | 77 | 19.6 | 23 | 14.3 | 32 | 20.9 | 0.74 (0.47, 1.18) | 0.66 (0.36, 1.22) |
| **CRNMB** |  |  |  |  |  |  |  |  |  |  |
| Any DOAC vs warfarin | 488 | 93.8 | 984 | 108.3 | 364 | 90.6 | 477 | 123.5 | 0.86 (0.77, 0.96) | 0.74 (0.66, 0.83) |
| Apixaban vs warfarin | 119 | 82.1 | 984 | 108.3 | 119 | 81.3 | 189 | 133.3 | 0.75 (0.62, 0.90) | 0.61 (0.51, 0.74) |
| Rivaroxaban vs warfarin | 353 | 98.0 | 984 | 108.3 | 353 | 97.1 | 424 | 117.3 | 0.90 (0.80, 1.02) | 0.82 (0.73, 0.91) |
| Apixaban vs rivaroxaban | 119 | 82.1 | 353 | 98.1 | 119 | 81.4 | 152 | 112.5 | 0.83 (0.67, 1.02) | 0.76 (0.58, 0.99) |
| **Clinically relevant bleeding** ^*^ |  |  |  |  |  |  |  |  |  |  |
| Any DOAC vs warfarin | 513 | 99.0 | 1064 | 117.7 | 383 | 95.3 | 503 | 130.2 | 0.84 (0.75, 0.93) | 0.74 (0.66, 0.82) |
| Apixaban vs warfarin | 125 | 86.2 | 1064 | 114.7 | 125 | 85.9 | 194 | 136.8 | 0.72 (0.60, 0.87) | 0.62 (0.52, 0.75) |
| Rivaroxaban vs warfarin | 371 | 103.1 | 1064 | 114.7 | 371 | 101.7 | 447 | 124.2 | 0.87 (0.78, 0.98) | 0.82 (0.74, 0.92) |
| Apixaban vs rivaroxaban | 125 | 86.2 | 371 | 103.1 | 125 | 84.7 | 161 | 117.9 | 0.83 (0.68, 1.01) | 0.74 (0.57, 0.97) |
| **Other composite outcomes** |  |  |  |  |  |  |  |  |  |  |
| **Recurrent VTE or clinically relevant bleeding** |  |  |  |  |  |  |  |  |  |  |
| Any DOAC vs warfarin | 542 | 105.0 | 1127 | 125.5 | 402 | 100.5 | 553 | 139.2 | 0.83 (0.75, 0.92) | 0.74 (0.66, 0.82) |
| Apixaban vs warfarin | 131 | 90.9 | 1127 | 125.5 | 131 | 89.1 | 204 | 148.2 | 0.71 (0.60, 0.86) | 0.63 (0.53, 0.75) |
| Rivaroxaban vs warfarin | 392 | 109.5 | 1127 | 125.5 | 392 | 109.2 | 470 | 132.4 | 0.88 (0.78, 0.98) | 0.82 (0.74, 0.91) |
| Apixaban vs rivaroxaban | 131 | 90.9 | 392 | 109.5 | 131 | 89.8 | 169 | 125.0 | 0.81 (0.67, 0.99) | 0.76 (0.58, 0.98) |
| **Any clinical outcome** |  |  |  |  |  |  |  |  |  |  |
| Any DOAC vs warfarin | 663 | 129.2 | 1308 | 146.3 | 495 | 126.2 | 612 | 159.7 | 0.88 (0.80, 0.96) | 0.79 (0.72, 0.87) |
| Apixaban vs warfarin | 178 | 124.5 | 1308 | 146.3 | 178 | 125.4 | 242 | 176.8 | 0.84 (0.72, 0.99) | 0.72 (0.62, 0.85) |
| Rivaroxaban vs warfarin | 465 | 130.6 | 1308 | 146.3 | 465 | 129.7 | 533 | 150.0 | 0.89 (0.80, 0.99) | 0.86 (0.78, 0.94) |
| Apixaban vs rivaroxaban | 178 | 124.5 | 465 | 130.6 | 178 | 129.3 | 215 | 163.6 | 0.95 (0.80, 1.13) | 0.84 (0.66, 1.05) |

^*^ The primary outcome measure was a composite of hospitalization for recurrent VTE and hospitalization for major bleeding. Clinically relevant bleeding was a composite of any bleeding event, whether major bleeding or clinically relevant bleeding. Abbreviations. CRNMB, clinically relevant non-major bleeding; DOACs, direct oral anticoagulants; hazards ratio, HR; IR, incidence rate; PY, person-years.

**Appendix Table 11: Incidence rates and effect estimates for clinical outcomes by treatment groups using an alternative twelve month follow-up period.**

| **Clinical outcomes** | **Before PSM** | | | | **After PSM** | | | | **Unmatched HR**  **(95% CI)** | **PSM HR**  **(95% CI)** |
| --- | --- | --- | --- | --- | --- | --- | --- | --- | --- | --- |
|  | **Comparator** | | **Reference** | | **Comparator** | | **Reference** | |  |  |
|  | **n** | **IR/100 PY** | **n** | **IR/100 PY** | **n** | **IR/100 PY** | **n** | **IR/100 PY** |  |  |
| **Primary outcome** |  |  |  |  |  |  |  |  |  |  |
| Any DOAC vs warfarin | 219 | 20.6 | 611 | 30.9 | 179 | 21.8 | 263 | 31.5 | 0.65 (0.55, 0.75) | 0.70 (0.60, 0.83) |
| Apixaban vs warfarin | 51 | 18.1 | 611 | 30.9 | 51 | 17.4 | 101 | 35.7 | 0.51 (0.41, 0.73) | 0.48 (0.36, 0.65) |
| Rivaroxaban vs warfarin | 161 | 21.3 | 611 | 30.9 | 161 | 20.7 | 230 | 30.4 | 0.68 (0.57, 0.81) | 0.69 (0.58, 0.82) |
| Apixaban vs rivaroxaban | 51 | 18.1 | 161 | 21.3 | 51 | 18.4 | 60 | 22.1 | 0.75 (0.54, 1.05) | 0.81 (0.59, 1.11) |
| **Effectiveness outcomes** |  |  |  |  |  |  |  |  |  |  |
| **Recurrent VTE** |  |  |  |  |  |  |  |  |  |  |
| Any DOAC vs warfarin | 65 | 6.0 | 156 | 7.6 | 53 | 7.1 | 69 | 9.0 | 0.76 (0.57, 1.02) | 0.77 (0.57, 1.03) |
| Apixaban vs warfarin | 16 | 5.7 | 156 | 7.6 | 16 | 5.7 | 21 | 13.8 | 0.69 (0.42, 1.16) | 0.44 (0.26, 0.75) |
| Rivaroxaban vs warfarin | 46 | 6.0 | 156 | 7.6 | 46 | 6.6 | 63 | 8.9 | 0.77 (0.55, 1.07) | 0.79 (0.57, 1.09) |
| Apixaban vs rivaroxaban | 16 | 5.7 | 46 | 6.0 | 16 | 6.1 | 21 | 7.9 | 0.92 (0.52, 1.62) | 0.81 (0.37, 1.76) |
| **All-cause mortality** |  |  |  |  |  |  |  |  |  |  |
| Any DOAC vs warfarin | 296 | 27.4 | 475 | 23.1 | 236 | 29.9 | 226 | 28.1 | 1.15 (0.98, 1.33) | 1.13 (0.97, 1.32) |
| Apixaban vs warfarin | 108 | 38.2 | 475 | 23.1 | 108 | 37.7 | 124 | 43.9 | 1.54 (1.25, 1.90) | 1.16 (0.92, 1.45) |
| Rivaroxaban vs warfarin | 182 | 23.7 | 475 | 23.1 | 182 | 23.9 | 193 | 25.5 | 1.01 (0.85, 1.20) | 0.99 (0.84, 1.19) |
| Apixaban vs rivaroxaban | 108 | 38.2 | 182 | 23.7 | 108 | 41.2 | 111 | 42.1 | 1.51 (1.18, 1.92) | 1.04 (0.75, 1.44) |
| **Safety outcomes** |  |  |  |  |  |  |  |  |  |  |
| **Major bleeding** |  |  |  |  |  |  |  |  |  |  |
| Any DOAC vs warfarin | 164 | 15.3 | 482 | 24.1 | 136 | 15.9 | 205 | 23.6 | 0.62 (0.52, 0.73) | 0.69 (0.58, 0.83) |
| Apixaban vs warfarin | 36 | 12.8 | 482 | 24.1 | 36 | 12.3 | 71 | 24.8 | 0.50 (0.35, 0.70) | 0.44 (0.31, 0.83) |
| Rivaroxaban vs warfarin | 124 | 16.3 | 482 | 24.1 | 124 | 15.1 | 170 | 20.2 | 0.67 (0.55, 0.81) | 0.92 (0.70, 1.20) |
| Apixaban vs rivaroxaban | 36 | 12.8 | 124 | 16.3 | 36 | 12.3 | 44 | 15.8 | 0.66 (0.45, 0.98) | 0.76 (0.44, 1.32) |
| **CRNMB** |  |  |  |  |  |  |  |  |  |  |
| Any DOAC vs warfarin | 800 | 84.9 | 1622 | 94.4 | 632 | 87.6 | 764 | 105.9 | 0.88 (0.81, 0.96) | 0.79 (0.71, 0.86) |
| Apixaban vs warfarin | 189 | 73.5 | 1622 | 94.4 | 189 | 72.7 | 291 | 116.2 | 0.75 (0.64, 0.87) | 0.62 (0.53, 0.73) |
| Rivaroxaban vs warfarin | 584 | 88.1 | 1622 | 94.4 | 584 | 87.6 | 673 | 101.8 | 0.92 (0.84, 1.02) | 0.85 (0.78, 0.94) |
| Apixaban vs rivaroxaban | 189 | 73.5 | 584 | 88.1 | 189 | 73.1 | 240 | 95.0 | 0.78 (0.66, 0.92) | 0.74 (0.58, 0.93) |
| **Clinically relevant bleeding** ^*^ |  |  |  |  |  |  |  |  |  |  |
| Any DOAC vs warfarin | 830 | 88.3 | 1744 | 102.3 | 659 | 91.8 | 804 | 111.3 | 0.84 (0.78, 0.92) | 0.79 (0.72, 0.86) |
| Apixaban vs warfarin | 196 | 76.9 | 1744 | 102.3 | 196 | 75.5 | 297 | 119.4 | 0.72 (0.62, 0.83) | 0.62 (0.53, 0.72) |
| Rivaroxaban vs warfarin | 606 | 91.7 | 1744 | 102.3 | 606 | 90.8 | 707 | 107.3 | 0.89 (0.81, 0.97) | 0.84 (0.77, 0.93) |
| Apixaban vs rivaroxaban | 196 | 76.9 | 606 | 91.7 | 196 | 75.7 | 250 | 99.0 | 0.78 (0.66, 0.92) | 0.73 (0.58, 0.92) |
| **Other composite outcomes** |  |  |  |  |  |  |  |  |  |  |
| **Recurrent VTE or clinically relevant bleeding** |  |  |  |  |  |  |  |  |  |  |
| Any DOAC vs warfarin | 869 | 93.1 | 1832 | 108.5 | 688 | 96.8 | 837 | 117.0 | 0.84 (0.77, 0.91) | 0.79 (0.72, 0.86) |
| Apixaban vs warfarin | 205 | 80.4 | 1832 | 108.5 | 205 | 79.1 | 313 | 126.5 | 0.71 (0.62, 0.82) | 0.62 (0.53, 0.72) |
| Rivaroxaban vs warfarin | 634 | 96.6 | 1832 | 108.5 | 634 | 96.4 | 738 | 112.5 | 0.88 (0.80, 0.96) | 0.84 (0.76, 0.92) |
| Apixaban vs rivaroxaban | 205 | 80.4 | 634 | 96.6 | 205 | 79.6 | 263 | 104.0 | 0.78 (0.67, 0.92) | 0.73 (0.58, 0.92) |
| **Any clinical outcome** |  |  |  |  |  |  |  |  |  |  |
| Any DOAC vs warfarin | 1061 | 114.1 | 2126 | 126.2 | 842 | 119.8 | 965 | 136.8 | 0.88 (0.82, 0.95) | 0.84 (0.77, 0.91) |
| Apixaban vs warfarin | 275 | 108.7 | 2126 | 126.2 | 275 | 106.4 | 386 | 157.7 | 0.83 (0.73, 0.94) | 0.71 (0.61, 0.81) |
| Rivaroxaban vs warfarin | 752 | 114.9 | 2126 | 126.2 | 752 | 114.1 | 851 | 130.6 | 0.90 (0.83, 0.98) | 0.87 (0.80, 0.95) |
| Apixaban vs rivaroxaban | 275 | 108.7 | 752 | 114.9 | 275 | 110.8 | 337 | 137.4 | 0.90 (0.78, 1.04) | 0.81 (0.66, 0.99) |

^*^ The primary outcome measure was a composite of hospitalization for recurrent VTE and hospitalization for major bleeding. Clinically relevant bleeding was a composite of any bleeding event, whether major bleeding or clinically relevant bleeding. Abbreviations. CRNMB, clinically relevant non-major bleeding; DOACs, direct oral anticoagulants; hazards ratio, HR; IR, incidence rate; PY, person-years.

**Appendix Table 12: Incidence rates and effect estimates for clinical outcomes by treatment groups using an intent-to-treat design.**

| **Clinical outcomes** | **Before PSM** | | | | **After PSM** | | | | **Unmatched HR**  **(95% CI)** | **PSM HR**  **(95% CI)** |
| --- | --- | --- | --- | --- | --- | --- | --- | --- | --- | --- |
|  | **Comparator** | | **Reference** | | **Comparator** | | **Reference** | |  |  |
|  | **n** | **IR/100 PY** | **n** | **IR/100 PY** | **n** | **IR/100 PY** | **n** | **IR/100 PY** |  |  |
| **Primary outcome** |  |  |  |  |  |  |  |  |  |  |
| Any DOAC vs warfarin | 202 | 12.3 | 550 | 18.3 | 166 | 13.4 | 236 | 18.3 | 0.65 (0.56, 0.77) | 0.74 (0.64, 0.86) |
| Apixaban vs warfarin | 46 | 10.6 | 550 | 18.3 | 46 | 10.2 | 86 | 20.6 | 0.54 (0.40, 0.74) | 0.53 (0.39, 0.71) |
| Rivaroxaban vs warfarin | 150 | 13.0 | 550 | 18.3 | 150 | 12.6 | 204 | 17.4 | 0.69 (0.58, 0.83) | 0.80 (0.68, 0.95) |
| Apixaban vs rivaroxaban | 46 | 10.6 | 150 | 13.0 | 46 | 10.3 | 58 | 14.1 | 0.78 (0.56, 1.09) | 0.69 (0.43, 1.09) |
| **Effectiveness outcomes** |  |  |  |  |  |  |  |  |  |  |
| **Recurrent VTE** |  |  |  |  |  |  |  |  |  |  |
| Any DOAC vs warfarin | 58 | 3.4 | 134 | 4.2 | 47 | 4.1 | 60 | 4.9 | 0.78 (0.57, 1.06) | 0.79 (0.59, 1.05) |
| Apixaban vs warfarin | 13 | 2.9 | 134 | 4.2 | 13 | 2.6 | 29 | 7.1 | 0.65 (0.36, 1.18) | 0.46 (0.26, 0.80) |
| Rivaroxaban vs warfarin | 42 | 3.5 | 134 | 4.2 | 42 | 3.9 | 55 | 4.8 | 0.81 (0.57, 1.14) | 0.84 (0.62, 1.15) |
| Apixaban vs rivaroxaban | 13 | 2.9 | 42 | 3.5 | 13 | 2.8 | 20 | 5.1 | 0.80 (0.43, 1.48) | 0.87 (0.38, 1.97) |
| **All-cause mortality** |  |  |  |  |  |  |  |  |  |  |
| Any DOAC vs warfarin | 270 | 16.0 | 435 | 13.7 | 213 | 17.7 | 204 | 16.3 | 1.14 (0.98, 1.33) | 1.17 (0.90, 1.36) |
| Apixaban vs warfarin | 101 | 23.0 | 435 | 13.7 | 101 | 22.9 | 107 | 26.1 | 1.59 (1.28, 1.99) | 1.03 (0.81, 1.24) |
| Rivaroxaban vs warfarin | 163 | 13.7 | 435 | 13.7 | 163 | 14.2 | 175 | 14.6 | 0.98 (0.82, 1.17) | 0.90 (0.76, 1.07) |
| Apixaban vs rivaroxaban | 101 | 23.0 | 163 | 13.7 | 101 | 25.1 | 99 | 24.7 | 1.64 (1.28, 2.10) | 1.05 (0.76, 1.46) |
| **Safety outcomes** |  |  |  |  |  |  |  |  |  |  |
| **Major bleeding** |  |  |  |  |  |  |  |  |  |  |
| Any DOAC vs warfarin | 153 | 9.3 | 441 | 14.4 | 128 | 9.9 | 186 | 13.8 | 0.62 (0.52, 0.75) | 0.72 (0.60, 0.86) |
| Apixaban vs warfarin | 34 | 7.8 | 441 | 14.4 | 34 | 7.5 | 63 | 14.0 | 0.51 (0.36, 0.72) | 0.53 (0.37, 0.73) |
| Rivaroxaban vs warfarin | 116 | 9.9 | 441 | 14.4 | 116 | 9.4 | 159 | 12.9 | 0.67 (0.55, 0.83) | 0.79 (0.65, 0.95) |
| Apixaban vs rivaroxaban | 34 | 7.8 | 116 | 9.9 | 34 | 7.4 | 43 | 9.8 | 0.75 (0.51, 1.10) | 0.58 (0.33, 1.01) |
| **CRNMB** |  |  |  |  |  |  |  |  |  |  |
| Any DOAC vs warfarin | 735 | 51.9 | 1497 | 58.4 | 579 | 54.7 | 718 | 65.9 | 0.87 (0.79, 0.95) | 0.80 (0.73, 0.88) |
| Apixaban vs warfarin | 178 | 46.4 | 1497 | 58.4 | 178 | 46.3 | 276 | 72.5 | 0.76 (0.65, 0.89) | 0.61 (0.52, 0.71) |
| Rivaroxaban vs warfarin | 532 | 53.5 | 1497 | 58.4 | 532 | 53.9 | 626 | 62.4 | 0.90 (0.82, 0.99) | 0.86 (0.78, 0.94) |
| Apixaban vs rivaroxaban | 178 | 46.4 | 532 | 53.5 | 178 | 46.1 | 225 | 62.3 | 0.84 (0.71, 1.00) | 0.71 (0.57, 0.90) |
| **Clinically relevant bleeding** ^*^ |  |  |  |  |  |  |  |  |  |  |
| Any DOAC vs warfarin | 767 | 54.6 | 1611 | 63.7 | 608 | 57.9 | 754 | 69.6 | 0.84 (0.77, 0.91) | 0.81 (0.74, 0.88) |
| Apixaban vs warfarin | 185 | 48.7 | 1611 | 63.7 | 185 | 47.9 | 282 | 75.0 | 0.73 (0.63, 0.85) | 0.62 (0.54, 0.73) |
| Rivaroxaban vs warfarin | 556 | 56.3 | 1611 | 63.7 | 556 | 56.6 | 656 | 66.0 | 0.87 (0.79, 0.96) | 0.84 (0.77, 0.93) |
| Apixaban vs rivaroxaban | 185 | 48.7 | 556 | 56.3 | 185 | 48.4 | 236 | 64.8 | 0.84 (0.71, 0.99) | 0.70 (0.55, 0.88) |
| **Other composite outcomes** |  |  |  |  |  |  |  |  |  |  |
| **Recurrent VTE or clinically relevant bleeding** |  |  |  |  |  |  |  |  |  |  |
| Any DOAC vs warfarin | 803 | 57.8 | 1685 | 67.5 | 635 | 61.2 | 782 | 73.4 | 0.84 (0.77, 0.91) | 0.79 (0.71, 0.88) |
| Apixaban vs warfarin | 192 | 50.8 | 1685 | 67.5 | 192 | 50.6 | 293 | 79.5 | 0.72 (0.62, 0.84) | 0.63 (0.54, 0.73) |
| Rivaroxaban vs warfarin | 583 | 59.8 | 1685 | 67.5 | 583 | 60.4 | 683 | 69.8 | 0.87 (0.79, 0.96) | 0.84 (0.77, 0.92) |
| Apixaban vs rivaroxaban | 192 | 50.8 | 583 | 59.8 | 192 | 50.7 | 248 | 69.9 | 0.83 (0.72, 0.97) | 0.72 (0.58, 0.93) |
| **Any clinical outcome** |  |  |  |  |  |  |  |  |  |  |
| Any DOAC vs warfarin | 979 | 71.3 | 1965 | 79.6 | 775 | 76.3 | 901 | 86.4 | 0.87 (0.81, 0.94) | 0.85 (0.79, 0.92) |
| Apixaban vs warfarin | 257 | 69.1 | 1965 | 79.6 | 257 | 68.8 | 363 | 100.7 | 0.83 (0.73, 0.95) | 0.69 (0.60, 0.79) |
| Rivaroxaban vs warfarin | 690 | 71.5 | 1965 | 79.6 | 690 | 72.2 | 788 | 81.3 | 0.88 (0.81, 0.96) | 0.87 (0.80, 0.94) |
| Apixaban vs rivaroxaban | 257 | 69.1 | 690 | 71.5 | 257 | 71.1 | 315 | 92.1 | 0.94 (0.82, 1.09) | 0.78 (0.64, 0.94) |

^*^ The primary outcome measure was a composite of hospitalization for recurrent VTE and hospitalization for major bleeding. Clinically relevant bleeding was a composite of any bleeding event, whether major bleeding or clinically relevant bleeding. Abbreviations. CRNMB, clinically relevant non-major bleeding; DOACs, direct oral anticoagulants; hazards ratio, HR; IR, incidence rate; PY, person-years.

**Appendix Table 13: Incidence rates and effect estimates for clinical outcomes by treatment groups after varying the maximum gap between prescription refills.**

|  | **Maximum gap of 7-days between prescriptions** | | **Maximum gap of 14-days between prescriptions** | | **Allowable gap between refills based on half-life of oral anticoagulants** | |
| --- | --- | --- | --- | --- | --- | --- |
| **Clinical outcomes** | **Unmatched HR (95% CI)** | **PSM HR (95% CI)** | **Unmatched HR (95% CI)** | **PSM HR (95% CI)** | **Unmatched HR (95% CI)** | **PSM HR (95% CI)** |
| **Primary outcome** |  |  |  |  |  |  |
| Any DOAC vs warfarin | 0.67 (0.56, 0.80) | 0.74 (0.62, 0.88) | 0.66 (0.56, 0.79) | 0.75 (0.63, 0.89) | 0.67 (0.57, 0.80) | 0.78 (0.66, 0.93) |
| Apixaban vs warfarin | 0.53 (0.39, 0.74) | 0.46 (0.33, 0.64) | 0.53 (0.38, 0.73) | 0.43 (0.31, 0.60) | 0.56 (0.41, 0.77) | 0.49 (0.35, 0.69) |
| Rivaroxaban vs warfarin | 0.73 (0.60, 0.88) | 0.78 (0.65, 0.94) | 0.72 (0.59, 0.87) | 0.74 (0.61, 0.89) | 0.72 (0.59, 0.87) | 0.80 (0.67, 0.97) |
| Apixaban vs rivaroxaban | 0.73 (0.51, 1.04) | 0.61 (0.35, 1.06) | 0.74 (0.52, 1.05) | 0.63 (0.35, 1.13) | 0.78 (0.54, 1.11) | 0.72 (0.48, 1.07) |
| **Effectiveness** |  |  |  |  |  |  |
| **Recurrent VTE** |  |  |  |  |  |  |
| Any DOAC vs warfarin | 0.77 (0.55, 1.07) | 0.78 (0.56, 1.09) | 0.76 (0.55, 1.06) | 0.79 (0.57, 1.09) | 0.77 (0.55, 1.07) | 0.97 (0.70, 1.35) |
| Apixaban vs warfarin | 0.66 (0.36, 1.19) | 0.53 (0.29, 0.96) | 0.65 (0.36, 1.18) | 0.50 (0.28, 0.91) | 0.68 (0.38, 1.23) | 0.53 (0.29, 0.96) |
| Rivaroxaban vs warfarin | 0.78 (0.53, 1.15) | 0.80 (0.56, 1.14) | 0.77 (0.53, 1.13) | 0.76 (0.53, 1.07) | 0.77 (0.52, 1.12) | 0.74 (0.52, 1.05) |
| Apixaban vs rivaroxaban | 0.85 (0.44, 1.64) | 0.91 (0.34, 2.48) | 0.85 (0.44, 1.65) | 0.82 (0.31, 2.17) | 0.89 (0.46, 1.72) | 0.80 (0.30, 2.14) |
| **All-cause mortality** |  |  |  |  |  |  |
| Any DOAC vs warfarin | 1.19 (1.01, 1.40) | 1.15 (0.96, 1.38) | 1.17 (0.99, 1.38) | 1.12 (0.94, 1.33) | 1.27 (1.08, 1.50) | 1.02 (0.82, 1.26) |
| Apixaban vs warfarin | 1.55 (1.23, 1.96) | 1.19 (0.93, 1.52) | 1.54 (1.21, 1.94) | 1.17 (0.91, 1.50) | 1.54 (1.21, 1.94) | 1.19 (0.93, 1.52) |
| Rivaroxaban vs warfarin | 1.06 (0.87, 1.29) | 1.11 (0.91, 1.36) | 1.04 (0.86, 1.27) | 1.11 (0.91, 1.35) | 1.04 (0.86, 1.26) | 1.13 (0.93, 1.38) |
| Apixaban vs rivaroxaban | 1.47 (1.13, 1.93) | 1.10 (0.70, 1.72) | 1.48 (1.13, 1.94) | 1.07 (0.68, 1.68) | 1.60 (1.22, 2.09) | 1.18 (0.83, 1.75) |
| **Safety** |  |  |  |  |  |  |
| **Major bleeding** |  |  |  |  |  |  |
| Any DOAC vs warfarin | 0.63 (0.52, 0.77) | 0.72 (0.59, 0.87) | 0.62 (0.51, 0.76) | 0.74 (0.61, 0.90) | 0.68 (0.55, 0.83) | 0.73 (0.61, 0.89) |
| Apixaban vs warfarin | 0.48 (0.33, 0.71) | 0.40 (0.26, 0.60) | 0.48 (0.33, 0.70) | 0.37 (0.25, 0.56) | 0.54 (0.37, 0.79) | 0.44 (0.30, 0.66) |
| Rivaroxaban vs warfarin | 0.71 (0.57, 0.88) | 0.79 (0.64, 0.98) | 0.70 (0.56, 0.87) | 0.75 (0.61, 0.93) | 0.70 (0.56, 0.87) | 0.83 (0.67, 1.03) |
| Apixaban vs rivaroxaban | 0.68 (0.45, 1.03) | 0.60 (0.35, 1.03) | 0.69 (0.45, 1.03) | 0.56 (0.27, 1.17) | 0.73 (0.48, 1.10) | 0.58 (0.30, 1.12) |
| **CRNMB** |  |  |  |  |  |  |
| Any DOAC vs warfarin | 0.89 (0.81, 0.98) | 0.82 (0.74, 0.91) | 0.87 (0.80, 0.96) | 0.81 (0.73, 0.89) | 0.90 (0.81, 0.98) | 0.86 (0.78, 0.96) |
| Apixaban vs warfarin | 0.77 (0.66, 0.91) | 0.64 (0.54, 0.76) | 0.76 (0.65, 0.90) | 0.60 (0.51, 0.71) | 0.76 (0.65, 0.90) | 0.57 (0.43, 0.76) |
| Rivaroxaban vs warfarin | 0.94 (0.84, 1.04) | 0.93 (0.73, 1.04) | 0.92 (0.83, 1.02) | 0.88 (0.80, 0.98) | 0.92 (0.83, 1.02) | 0.91 (0.82, 1.01) |
| Apixaban vs rivaroxaban | 0.83 (0.69, 0.98) | 0.74 (0.57, 1.02) | 0.83 (0.69, 0.99) | 0.73 (0.55, 0.96) | 0.88 (0.74, 1.06) | 0.77 (0.60, 1.03) |
| **Clinically relevant bleeding** ^*^ |  |  |  |  |  |  |
| Any DOAC vs warfarin | 0.86 (0.79, 0.95) | 0.82 (0.74, 0.91) | 0.85 (0.78, 0.93) | 0.81 (0.74, 0.90) | 0.87 (0.79, 0.95) | 0.86 (0.76, 0.96) |
| Apixaban vs warfarin | 0.75 (0.64, 0.88) | 0.64 (0.54, 0.76) | 0.74 (0.63, 0.87) | 0.60 (0.51, 0.71) | 0.74 (0.63, 0.87) | 0.71 (0.60, 0.84) |
| Rivaroxaban vs warfarin | 0.91 (0.82, 1.01) | 0.94 (0.84, 1.04) | 0.89 (0.80, 0.99) | 0.88 (0.80, 0.98) | 0.89 (0.81, 0.99) | 0.92 (0.83, 1.01) |
| Apixaban vs rivaroxaban | 0.82 (0.68, 0.98) | 0.77 (0.58, 1.01) | 0.83 (0.69, 0.99) | 0.85 (0.78, 0.93) | 0.88 (0.74, 1.05) | 0.83 (0.65, 1.06) |
| **Other composite outcomes** |  |  |  |  |  |  |
| **Recurrent VTE or clinically relevant bleeding** |  |  |  |  |  |  |
| Any DOAC vs warfarin | 0.86 (0.79, 0.94) | 0.82 (0.75, 0.90) | 0.85 (0.78, 0.93) | 0.82 (0.74, 0.90) | 0.92 (0.84, 1.01) | 0.87 (0.79, 0.96) |
| Apixaban vs warfarin | 0.74 (0.63, 0.87) | 0.65 (0.55, 0.77) | 0.73 (0.62, 0.85) | 0.61 (0.51, 0.72) | 0.73 (0.62, 0.85) | 0.71 (0.61, 0.84) |
| Rivaroxaban vs warfarin | 0.91 (0.82, 1.01) | 0.92 (0.83, 1.02) | 0.89 (0.80, 0.98) | 0.78 (0.68, 0.90) | 0.89 (0.80, 0.98) | 0.90 (0.81, 0.99) |
| Apixaban vs rivaroxaban | 0.82 (0.69, 0.97) | 0.75 (0.58, 0.98) | 0.82 (0.69, 0.97) | 0.74 (0.57, 0.96) | 0.87 (0.73, 1.04) | 0.80 (0.62, 1.05) |
| **Any clinical outcome** |  |  |  |  |  |  |
| Any DOAC vs warfarin | 0.91 (0.84, 0.99) | 0.88 (0.81, 0.96) | 0.90 (0.83, 0.97) | 0.87 (0.80, 0.95) | 0.97 (0.90, 1.05) | 0.94 (0.86, 1.03) |
| Apixaban vs warfarin | 0.86 (0.75, 0.98) | 0.74 (0.63, 0.86) | 0.84 (0.74, 0.97) | 0.71 (0.61, 0.82) | 0.84 (0.74, 0.97) | 0.80 (0.69, 0.92) |
| Rivaroxaban vs warfarin | 0.93 (0.85, 1.02) | 0.95 (0.86, 1.04) | 0.91 (0.83, 1.00) | 0.90 (0.83, 0.99) | 0.91 (0.83, 1.00) | 0.94 (0.86, 1.03) |
| Apixaban vs rivaroxaban | 0.92 (0.79, 1.07) | 0.81 (0.64, 1.02) | 0.92 (0.80, 1.08) | 0.80 (0.63, 1.01) | 0.99 (0.85, 1.15) | 0.86 (0.67, 1.08) |

^*^ The primary outcome measure was a composite of hospitalization for recurrent VTE and hospitalization for major bleeding. Clinically relevant bleeding was a composite of any bleeding event, whether major bleeding or clinically relevant bleeding. Abbreviations. CRNMB, clinically relevant non-major bleeding; DOACs, direct oral anticoagulants; hazards ratio, HR; IR, incidence rate; PY, person-years.

**Appendix Table 14: Incidence rates and effect estimates for clinical outcomes in cohort restricted to patients that initiated oral anticoagulation between January 1, 2011, and June 30, 2017**

| **Clinical outcomes** | **Before PSM** | | | | **After PSM** | | | | **Unmatched HR**  **(95% CI)** | **PSM HR**  **(95% CI)** |
| --- | --- | --- | --- | --- | --- | --- | --- | --- | --- | --- |
|  | **DOACs**  **(n=2557; 33.3%)** | | **Warfarin**  **(n=5134; 66.7%)** | | **DOACs**  **(n = 2110; 50%)** | | **Warfarin**  **(n = 2110; 50%)** | |  |  |
|  | **n** | **IR/100 PY** | **n** | **IR/100 PY** | **n** | **IR/100 PY** | **n** | **IR/100 PY** |  |  |
| **Primary outcome** | 145 | 19.4 | 465 | 30.9 | 121 | 19.2 | 191 | 31.8 | 0.63 (0.52, 0.76) | 0.61 (0.51, 0.74) |
| **Effectiveness outcomes** |  |  |  |  |  |  |  |  |  |  |
| Recurrent VTE | 37 | 4.9 | 112 | 7.2 | 26 | 4.3 | 48 | 8.5 | 0.67 (0.47, 0.98) | 0.50 (0.34, 0.74) |
| Death | 193 | 25.5 | 350 | 22.5 | 155 | 26.7 | 145 | 25.9 | 1.14 (0.95, 1.36) | 1.15 (0.96, 1.38) |
| **Safety outcomes** |  |  |  |  |  |  |  |  |  |  |
| Major bleeding | 113 | 15.0 | 375 | 24.7 | 99 | 15.3 | 152 | 24.4 | 0.61 (0.49, 0.75) | 0.63 (0.51, 0.78) |
| CRNMB | 550 | 81.4 | 1264 | 94.3 | 459 | 83.3 | 557 | 103.6 | 0.87 (0.79, 0.96) | 0.81 (0.73, 0.90) |
| Clinically relevant bleeding | 578 | 85.8 | 1363 | 102.4 | 483 | 88.1 | 583 | 108.9 | 0.84 (0.77, 0.93) | 0.82 (0.74, 0.91) |
| **Other composite outcomes** |  |  |  |  |  |  |  |  |  |  |
| Recurrent VTE or Clinically relevant bleeding | 602 | 89.9 | 1428 | 108.2 | 501 | 91.9 | 608 | 114.3 | 0.84 (0.76, 0.92) | 0.82 (0.74, 0.91) |
| Any clinical outcome | 740 | 110.9 | 1662 | 126.3 | 608 | 112.7 | 694 | 132.5 | 0.88 (0.81, 0.95) | 0.86 (0.79, 0.95) |

The primary outcome measure was a composite of hospitalization for recurrent VTE and hospitalization for major bleeding. Abbreviations. CRNMB, clinically relevant non-major bleeding; DOACs, direct oral anticoagulants; HR, hazards ratio; IR, incidence rate; PSM, propensity-score matching; PY, person-years; VTE, venous thromboembolism.

References

1. Ballestri S, Capitelli M, Fontana MC, et al. Direct Oral Anticoagulants in Patients with Liver Disease in the Era of Non-Alcoholic Fatty Liver Disease Global Epidemic: A Narrative Review. *Adv Ther*. 2020;37(5):1910-1932. doi:10.1007/s12325-020-01307-z

2. Qamar A, Vaduganathan M, Greenberger NJ, Giugliano RP. Oral Anticoagulation in Patients With Liver Disease. *J Am Coll Cardiol*. 2018;71(19):2162-2175. doi:10.1016/j.jacc.2018.03.023

3. Rakoski MO, McCammon RJ, Piette JD, et al. Burden of cirrhosis on older Americans and their families: analysis of the health and retirement study. *Hepatology*. 2012;55(1):184-191. doi:10.1002/hep.24616

4. Nehra MS, Ma Y, Clark C, Amarasingham R, Rockey DC, Singal AG. Use of administrative claims data for identifying patients with cirrhosis. *J Clin Gastroenterol*. 2013;47(5):e50-54. doi:10.1097/MCG.0b013e3182688d2f

5. Lo Re V, Lim JK, Goetz MB, et al. Validity of diagnostic codes and liver-related laboratory abnormalities to identify hepatic decompensation events in the Veterans Aging Cohort Study. *Pharmacoepidemiol Drug Saf*. 2011;20(7):689-699. doi:10.1002/pds.2148

6. White RH, Garcia M, Sadeghi B, et al. Evaluation of the predictive value of ICD-9-CM coded administrative data for venous thromboembolism in the United States. *Thromb Res*. 2010;126(1):61-67. doi:10.1016/j.thromres.2010.03.009

7. Sanfilippo KM, Wang TF, Gage BF, Liu W, Carson KR. Improving accuracy of International Classification of Diseases codes for venous thromboembolism in administrative data. *Thromb Res*. 2015;135(4):616-620. doi:10.1016/j.thromres.2015.01.012

8. Tamariz L, Harkins T, Nair V. A systematic review of validated methods for identifying venous thromboembolism using administrative and claims data. *Pharmacoepidemiol Drug Saf*. 2012;21 Suppl 1:154-162. doi:10.1002/pds.2341

9. Hagström H, Adams LA, Allen AM, et al. Administrative Coding in Electronic Health Care Record-Based Research of NAFLD: An Expert Panel Consensus Statement. *Hepatology*. 2021;74(1):474-482. doi:10.1002/hep.31726

10. Allen AM, Therneau TM, Larson JJ, Coward A, Somers VK, Kamath PS. Nonalcoholic Fatty Liver Disease Incidence and Impact on Metabolic Burden and Death: a 20 Year-Community Study. *Hepatology*. 2018;67(5):1726-1736. doi:10.1002/hep.29546

11. Cunningham A, Stein CM, Chung CP, Daugherty JR, Smalley WE, Ray WA. An automated database case definition for serious bleeding related to oral anticoagulant use. *Pharmacoepidemiology and Drug Safety*. 2011;20(6):560-566. doi:10.1002/pds.2109

12. Fralick M, Colacci M, Schneeweiss S, Huybrechts KF, Lin KJ, Gagne JJ. Effectiveness and Safety of Apixaban Compared With Rivaroxaban for Patients With Atrial Fibrillation in Routine Practice: A Cohort Study. *Ann Intern Med*. 2020;172(7):463-473. doi:10.7326/M19-2522

13. Shehab N, Ziemba R, Campbell KN, et al. Assessment of ICD-10-CM code assignment validity for case finding of outpatient anticoagulant-related bleeding among Medicare beneficiaries. *Pharmacoepidemiology and Drug Safety*. 2019;28(7):951-964. doi:10.1002/pds.4783

14. Kearon C, Ageno W, Cannegieter SC, et al. Categorization of patients as having provoked or unprovoked venous thromboembolism: guidance from the SSC of ISTH. *J Thromb Haemost*. 2016;14(7):1480-1483. doi:10.1111/jth.13336

15. Center for Drug Evaluation and Research. Drug Development and Drug Interactions | Table of Substrates, Inhibitors and Inducers. *FDA*. Published online May 26, 2021. Accessed December 4, 2021. https://www.fda.gov/drugs/drug-interactions-labeling/drug-development-and-drug-interactions-table-substrates-inhibitors-and-inducers

16. Steffel J, Verhamme P, Potpara TS, et al. The 2018 European Heart Rhythm Association Practical Guide on the use of non-vitamin K antagonist oral anticoagulants in patients with atrial fibrillation. *Eur Heart J*. 2018;39(16):1330-1393. doi:10.1093/eurheartj/ehy136

17. Pisters R, Lane DA, Nieuwlaat R, de Vos CB, Crijns HJGM, Lip GYH. A novel user-friendly score (HAS-BLED) to assess 1-year risk of major bleeding in patients with atrial fibrillation: the Euro Heart Survey. *Chest*. 2010;138(5):1093-1100. doi:10.1378/chest.10-0134

18. Kim DH, Schneeweiss S, Glynn RJ, Lipsitz LA, Rockwood K, Avorn J. Measuring Frailty in Medicare Data: Development and Validation of a Claims-Based Frailty Index. *J Gerontol A Biol Sci Med Sci*. 2018;73(7):980-987. doi:10.1093/gerona/glx229
